# Unexpected Molecular Sieving of Xylene Isomer Using Tethered Ligand in Polymer‐Metal–Organic Frameworks (polyMOFs)

**DOI:** 10.1002/advs.202402980

**Published:** 2024-07-08

**Authors:** Taehoon Hyun, Junkil Park, Jungseob So, Jihan Kim, Dong‐Yeun Koh

**Affiliations:** ^1^ Department of Chemical and Biomolecular Engineering (BK‐21 Plus) Korea Advanced Institute of Science and Technology Daejeon 34141 South Korea; ^2^ Environment & Sustainable Resources Research Center Korea Research Institute of Chemical Technology Daejeon 34114 South Korea; ^3^ Saudi Aramco‐KAIST CO2 Management Center Korea Advanced Institute of Science and Technology Daejeon 34141 South Korea

**Keywords:** MOF‐polymer hybrid material, molecular sieving, polyMOFs, pore engineering

## Abstract

Promising advances in adsorption technology can lead to energy‐efficient solutions in industrial sectors. This work presents precise molecular sieving of xylene isomers in the polymer‐metal‐oragnic framework (polyMOF), a hybrid porous material derived from the parent isoreticular MOF‐1 (IRMOF‐1). PolyMOFs are synthesized by polymeric ligands bridged by evenly spaced alkyl chains, showing reduced pore sizes and enhanced stabilities compared to its parent material due to tethered polymer bridge within the pores while maintaining the original rigid crystal lattice. However, the exact configuration of the ligands within the crystals remain unclear, posing hurdles to predicting the adsorption performances of the polyMOFs. This work reveals that the unique pore structure of polyIRMOF‐1‐7a can discriminate xylene isomers with sub‐angstrom size differences, leading to highly selective adsorption of p‐xylene over other isomers and alkylbenzenes in complex liquid mixtures (α_pX/OM_ = 15 and α_pX/OME_ = 9). The structural details of the polyIRMOF‐1‐7a are elucidated through computational studies, suggesting a plausible configuration of alkyl chains within the polyMOF crystal, which enable a record‐high p‐xylene selectivity and stability in liquid hydrocarbon. With this unprecedented molecular selectivity in MOFs, “polymer‐MOF” hybridization is expected to meet rigorous requirements for high‐standard molecular sieving through precisely tunable and highly stable pores.

## Graphical abstract

This research demonstrates the potential of adsorption technology using polyMOFs, a hybrid material between MOFs and polymer. By incorporating polymer ligands into MOF crystals, it achieves enhanced stability and precise molecular sieving of xylene isomers. The unique pore structure of polyIRMOF‐1‐7a enables highly selective adsorption of p‐xylene over other isomers, achieving energy‐efficient adsorptive separation.

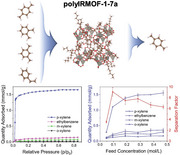

## 1 Introduction

Large‐scale separation of valuable chemical feedstock through thermally‐induced phase transition has been extensively practiced worldwide. These bulk phase transition procedures contribute 10–15% of the world's total energy consumption and account for up to 70% of chemical plant operational costs.^[^ ^1^, ^2^ ^]^ The predominant energy source for industrial separation processes stems from hydrocarbon resources, resulting in significant CO_2_ emissions. Current efforts are focused on developing substitute energy‐efficient separation technologies for traditional “thermal” separations, aiming to achieve dramatic reductions in energy and carbon intensity.^[^ ^1^, ^3^ ^]^ The separation of xylene isomers, critical precursors for commodities, has been considered one of the most challenging separation processes due to their similar physical properties (e.g., boiling point, molecular weight, and kinetic diameters; see Table S1, Supporting Information for physical properties).^[^ ^4^ ^]^ Given the difficulty of achieving xylene isomer separation through distillation, industrial processes have opted for fractional crystallization and adsorptive separation as alternative methods.^[^ ^5^ ^]^ Since the crystallization process inevitably accompanies a large amount of energy input and limited product purity, the simulated moving bed (SMB) process was dominantly adopted in industries such as UOP's Parex, Toray's Aromax, and IFP's Eluxyl.^[^ ^6^ ^]^ The selection of desirable adsorbent was one of the most critical factors for determining the whole separation efficiency of the SMB process. Zeolites have been mainly utilized as a desirable sorbent for SMB processes due to their rigid micropores, high surface area, thermal stability, and affinities for hydrocarbon molecules.^[^ ^7^, ^8^, ^9^, ^10^, ^11^ ^]^ The primary governing factor in separating xylene isomers within zeolites has involved the interaction between the framework cation and the aromatic ring of xylene, along with the packing of xylene molecules in the micropores.^[^ ^12^, ^13^ ^]^ However, due to the similar interactions across all xylene isomers, attaining high adsorption selectivity has proven challenging for zeolites. Significant endeavors have been focused on refining the pore size and acidity of zeolites by substituting framework cations to enhance the separation efficiency.^[^ ^14^, ^15^ ^]^ However, the topological restriction and limited selectivity of zeolites still remain a challenge.

Over the past decade, intrinsically porous materials, including metal–organic framework (MOF),^[^ ^16^, ^17^, ^18^, ^19^, ^20^, ^21^ ^]^ covalent‐organic framework (COF),^[^ ^22^ ^]^ organic cages,^[^ ^23^ ^]^ and coordination polymers,^[^ ^24^ ^]^ have been explored as suitable materials for separation of xylene isomers. Among the potential sorbents, metal–organic frameworks (MOFs) have been extensively studied due to their diverse pore geometries and functional moieties by combining different metal elements and ligands. Within the MOFs, the isolation of p‐xylene from the C_8_ aromatics blend can be accomplished through selective adsorption or exclusion. Considering the limited presence of p‐xylene (20–25 mol.%) in the realistic condition, a more energy‐efficient strategy would involve pursuing MOFs that selectively adsorb p‐xylene.^[^ ^25^ ^]^ Molecular discrimination of p‐xylene in micropores can be achieved through 1) efficient packing of p‐xylene within confined channels through entropic effects or 2) molecular sieving through size‐matched micropores. The prior strategy could be much more feasible, as it has the potential to most effectively exploit the distinctions between p‐xylene and the other isomers, specifically molecular structure symmetry.^[^ ^26^, ^27^, ^28^, ^29^ ^]^ Nonetheless, the appreciable adsorption of different isomers presented a challenge in achieving substantial p‐xylene selectivity. A limited number of MOFs exhibited the size‐dependent sieving effect over different xylene isomers (o‐xylene and m‐xylene). However, their selectivity remained unverified for discriminating p‐xylene from a realistic mixture that inevitably involves ethylbenzene.^[^ ^30^, ^31^ ^]^ Ethylbenzene has the same kinetic diameter as p‐xylene with one missing methyl group in the para‐position, making it difficult to discriminate from p‐xylene. Similar to zeolites, the ongoing development of novel MOFs is required to demonstrate enhanced selectivity toward p‐xylene across diverse mixture compositions.

**Figure 1:**
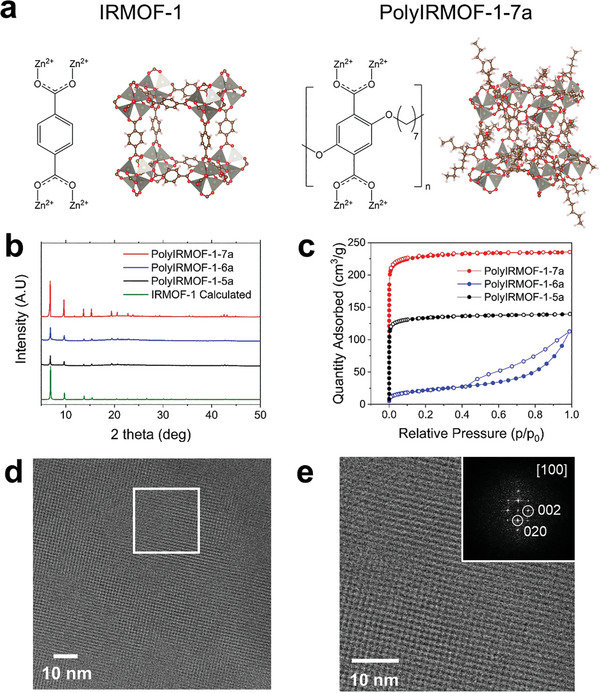
Characterization of polyIRMOFs: a) Molecular configuration of IRMOF‐1 and polyIRMOF‐1‐7a, b) powder x‐ray diffraction data, c) 77K nitrogen physisorption data of polyIRMOF‐1‐xa and high‐resolution TEM images of polyIRMOF‐1‐7a: d) surface of polyIRMOF‐1‐7a and e) magnified image in white rectangle in d). Inset shows a diffraction pattern of polyIRMOF‐1‐7a crystals, corresponding to the [100] zone axis orientation of polyIRMOF‐1‐7a, as IRMOF‐1.

Herein, we demonstrate that the polymer‐metal–organic framework (polyMOF) can act as a selective adsorbent for separating xylene isomers. The Cohen group first conceived the idea that, instead of using discrete ligand molecules, ligands bridged with polymer chains could be used as building blocks for MOFs (**Figure** **1**a).^[^ ^32^ ^]^ These polymer ligands can hinder the self‐ordering process of crystallization through chain entanglement and increased viscosity^[^ ^33^ ^]^; nevertheless, a series of polyMOFs were reported to be successfully synthesized and showed high crystalline structure identical to its parent MOF, isoreticular MOF‐1 (IRMOF‐1).^[^ ^32^, ^34^ ^]^ Successful synthesis of novel polyMOFs demonstrated its applicability beyond the primary IRMOF‐1 structure to encompass more intricate structures such as University of Oslo‐66 (UiO‐66) structure or PolyMOF based on polymers of intrinsic microporosity (PIMs).^[^ ^35^, ^36^ ^]^ Numerous advantages can be obtained through the utilization of polymer ligands; however, the primary emphasis was that the length of the bridging linker in polymeric ligands can be precisely controlled by using dibromoalkanes with various lengths (C_5_ through C_8_).^[^ ^32^ ^]^ These bridging chains will reside in the pores and can create a new pore environment by partitioning the pores or pore windows. It was confirmed that the polyMOFs generally show shrunk pore dimensions compared to its parent crystal, mostly due to the presence of the polymer ligands. However, the exact configuration of the alkyl chain within the formed polyMOFs remained unclear, due to the difficulties in detecting soft, flexible matters in confined pores via conventional spectroscopic measurements or computational methods. Additionally, there exists a notable gap in research concerning the application of polyMOFs for adsorptive separation despite their distinctive pore environments characterized by anchored alkyl chains. This absence of investigation underscores an untapped potential for exploring the efficacy of polyMOFs in the realm of separation science.

In this work, we report the unexpected size‐dependent molecular sieving of xylene isomers in polyIRMOF‐1‐7a, which was not observed in the parent IRMOF‐1 or other polyMOF derivatives. Our work proves that the inserted alkyl chain in the polyMOF would act as a nanoscopic barrier for molecular adsorption and diffusion. Computational studies revealed that the skewed position of the bridging alkyl chains penetrating the pore might induce the sharp molecular separation between p‐xylene and other benzene derivatives. The polyIRMOF‐1‐7a demonstrated exceptional p‐xylene adsorptive selectivity toward different xylene isomers (α_pX/OM_ = 12) for ternary liquid mixtures and even for quaternary liquid mixtures, including the cross‐effects from ethylbenzene (α_pX/OME_ = 9.09) at room temperature, rendering them more efficient for the energy‐intensive xylene separation.

## 2 Results and Discussion

### 2.1 Material Synthesis and Characterization

To compare the effect of linker length on the pore size, three different polymer ligands, pbdc‐xe (polymeric‐bdc‐ester, bdc = 1,4‐benzenedicarboxylic acid, x = 5, 6, and 7) were prepared by the step‐growth polymerization described in the literature.^[^ ^34^ ^]^ Term “x” denotes the number of ‐CH_2_‐ group between H_2_bdc units and can be controlled by using the dibromoalkane Br(CH_2_)_x_Br with different length (x = 5 to 7) in the polymerization step. Obtained polyethers were successively hydrolyzed to pbdc‐xa (polymeric‐bdc‐acid). After polymerization, the O‐H stretching peak at 3250 cm^−1^ of diethyl 2,5‐dihydroxyterephthalate disappeared in the Fourier‐transform infrared (FT‐IR) spectra of pbdc‐xe, indicating successful bridging of alkyl chain between bdc units (Figure S1, Supporting Information). Broad O‐H stretching peak (3300–2500 cm^−1^) of carboxylic acid in pbdc‐xa also appeared after hydrolysis of pbdc‐xe. Furthermore, ^1^H solution nuclear magnetic resonance (NMR) spectra results confirmed that peaks of the diethyl group (4.17 and 1.30 ppm) in diethyl 2,5‐dihydroxyterephthalate disappeared and peaks of bridged alkyl chain appeared after polymerization (Figure S2, Supporting Information).^[^ ^32^ ^]^

Each polymeric ligand was converted into polyMOFs with an IRMOF‐like network, designated as polyIRMOF‐1‐xa (x = 5 to 7), through the reaction with a zinc source. It was confirmed through powder x‐ray diffraction (PXRD) that all polyIRMOF‐1‐xa exhibited identical crystallinity to IRMOF‐1 (Figure 1b). Notably, although the bdc ligands were bridged by methylene linkers with different lengths, no significant difference exists in the crystal structures of polyIRMOF‐1‐xa compared to IRMOF‐1. Scanning electron microscope (SEM) images were obtained to observe the crystal morphology of polyIRMOF‐1‐xa (Figure S3, Supporting Information). Inter‐grown crystals of polyIRMOF‐1‐xa were known to originate from the sharing of polymer ligands during the growth of polyMOF crystals.^[^ ^32^ ^]^ While most polyIRMOF‐1‐5a crystals had spherical shapes, cubic morphology became clear with an increase in the number of alkyl spacers (from 5 to 7). Given that the IRMOF‐1 has a cubic structure, the sharp XRD pattern of polyIRMOF‐1‐7a, among other polyIRMOF derivatives, can be explained with enhanced crystallinity (Figure 1b). Interestingly, except for polyIRMOF‐1‐6a, both polyIRMOF‐1‐5a and polyIRMOF‐1‐7a showed type 1 isotherm (Figure 1c) for 77K N_2_ adsorption with Brunauer–Emmett–Teller (BET) surface area of 532 and 918 m^2^ g^−1^, respectively. Clarification was needed for the effect of linker length on the crystallinity and internal porosity of the polyIRMOF derivatives. Previous work suggested possible linkage configurations through density functional theory (DFT) calculation.^[^ ^34^ ^]^ Since the energy level variance was intensified with a linker with shorter alkyl spacers, a longer linker can make an energetically favorable configuration within the confined crystalline structure.^[^ ^34^ ^]^ However, further investigation is required to comprehend the synthetic mechanism of polyMOFs thoroughly.

Computational methods were employed in this work to better understand the ligand‐infiltrated pore structure of polyMOFs. As mentioned later, since both polyIRMOF‐1‐5a and polyIRMOF‐1‐6a showed non‐selective vapor adsorption for xylenes, the molecular structure modeling exclusively focused on polyIRMOF‐1‐7a. For the computational verification of the polyIRMOF‐1‐7a, first, all possible alkyl chain configurations bridging two bdc ligands in the unit cell were identified (‘configuration 1 to 4′ in Figure S4, Supporting Information). The stability of each configuration was compared by calculating their relative energy using the density functional theory (DFT) simulations (Supplementary Information, Computational Methods). According to the result, ‘configuration 2′ was found to be the most stable arrangement of alkyl chains, with minimum energy, consistent with the previous study.^[^ ^34^ ^]^ However, it is noteworthy that ‘configuration 3′ also exhibited structural stability with an energy difference of only 5 kJ mol^−1^ compared to ‘configuration 2′ (Figure S4, Supporting Information). Subsequently, pure IRMOF‐1 and polyIRMOF‐1‐7a structures were precisely reconstructed, with all pbdc ligands connected via alkyl chains in ‘configuration 2′ and ‘configuration 3′, respectively. The atomic ratio in the simulated structures closely matched the experimental elemental analysis results, indicating that the bdc ligands in polyIRMOF‐1‐7a were linked as one of these configurations (Table S2, Supporting Information). To explicitly examine the polymer ligand configurations in polyIRMOF‐1‐7a, high‐resolution cryogenic transmission electron microscope (cryoTEM) images were obtained (Supplementary Information, Materials and Methods). Despite employing the polymeric ligands, the cubic lattice structure of polyIRMOF‐1‐7a was confirmed, as observed in IRMOF‐1, however, the specific configuration of the ligand remained unverified through the imaging technique (Figure 1d).^[^ ^37^ ^]^ Since the direct identification of the ligand configuration is not feasible, textural properties were further compared to provide insight into the molecular configuration of polymeric ligands in polyIRMOF‐1‐7a. The simulated pore volume of pristine IRMOF‐1 (1.34 cm^3^ g^−1^) was in good agreement with the experimental t‐plot micropore volume (1.48 cm^3^ g^−1^) (Table S3, Supporting Information). Notably, for polyIRMOF‐1‐7a, the computationally prepared structure with all alkyl chains located in ‘configuration 2′ (Figure S5b, Supporting Information) exhibited a pore volume of 0.51 cm^3^ g^−1^, which largely deviated from the experimentally measured value of 0.36 cm^3^ g^−1^ by 29%. On the other hand, for ‘configuration 3′, the simulated pore volume was calculated as 0.36 cm^3^ g^−1^, matching the experimentally measured t‐plot micropore volume of polyIRMOF‐1‐7a. The relatively high simulated pore volume of ‘configuration 2′ originates from the substantial vacancy at the center of the unit cell (Figure S5b, Supporting Information). Conversely, the reduced pore volume of ‘configuration 3′ is a consequence of the reduced vacancy at the unit cell center caused by the alkyl chain connecting the bdc ligands in a crossed position (Figure S5c), penetrating the pores. Based on the assessment of pore volume, bridging alkyl chains in polyIRMOF‐1‐7‐a have a higher chance of being placed in ‘configuration 3′ rather than ‘configuration 2′. This suggests that a significant portion of the alkyl chain traverses the cavities of polyIRMOF‐1‐7a, leading to a substantial reduction in pore volume compared to its parent MOF and would eventually function as a nanoscopic partition for molecular sieving in the polyMOF.

To further compare the effect of the number of repeating units in bdc ligands on pore size and volume, we synthesized heptamethylene (C_7_) bridged linker (≈460 g mol^−1^), which have two bdc ligands and one C_7_ spacer between them (Compound 3 in Figure S6, Supporting Information). Using the single bridged linker, we crystallized the polyMOF (denoted as IRMOF‐1‐C_7_, Supplementary Information) using the same method and confirmed identical crystal structure with its parent MOF and polyIRMOF‐1‐7a (Figures S7 and S8, Supporting Information). The BET surface area (1910 m^2^ g^−1^) of the IRMOF‐1‐C_7_ was in between those of IRMOF‐1 (3800 m^2^ g^−1^) and polyIRMOF‐1‐7a (918 m^2^ g^−1^) (Figure S9, Table S3, Supporting Information). The micropore volume of IRMOF‐1‐C_7_ (0.74 cm^3^ g^−1^) was similar to that of the computationally modeled pore volume of ‘configuration 2′ in polyIRMOF‐1‐7a with <14% errors. Therefore, the IRMOF‐1‐C_7_ structure was more stable in ‘configuration 2′ of alkyl bridges. The MOF crystals synthesized from single bridged C_7_ ligands (≈460 g mol^−1^) showed less reduced pore volume compared to repeating polymer ligands used to crystalized polyIRMOF‐1‐7a, which had number averaged molecular weight of 5900 g mol^−1^ (see Table S3, Supporting Information).^[^ ^32^ ^]^ A similar effect was also observed when comparing the pore size distribution of the three types of crystals synthesized in this work—IRMOF‐1, IRMOF‐1‐C_7_, and polyIRMOF‐1‐7a. As shown in Figure S11 (Supporting Information), polyIRMOF‐1‐7a showed the smallest median pore width of 5.8 Å calculated with the Horvath–Kawazoe (HK) method. Density functional theory (DFT) was further employed to match the experimentally measured pore size distribution (PSD) with crystal structures. The pore size distribution (PSD) of polyIRMOF‐1‐7a derived from the HK method was closely matched with the PSD of the hypothetical structure formed with ‘configuration 3′ (Figures S11 and S12, Supporting Information). In the case of the simulated MOF structure with ‘configuration 2′, the DFT calculated PSD was predominantly concentrated within the range over 1 nm, primarily attributed to a non‐disturbed pore in the MOF structure (Figure S5b). The simulated PSD for IRMOF‐1‐C_7_ also showed pores larger than 1 nm were predominated, similar to the structure of polyIRMOF‐1‐7a with ‘configuration 2′ (Figure S12, Supporting Information). The textural properties of the three different types of MOFs in this study suggest that the substantial fraction of the bridging alkyl chain might have traversed the pore space in the polyIRMOF‐1‐7a, as suggested by the ‘configuration 3′ structure, resulting in a notable decrease in pore width and pore volume (Tables S2 and S3, Figure S12, Supporting Information). Considering that the kinetic diameters of the benzene derivatives range from 5.8 to 6.8 Å, pore‐penetrated polyIRMOF‐1‐7a can work as a molecular sieve for the sharp separation of molecules matching the range.

### 2.2 Unary Xylene Uptake Performance

**Figure 2:**
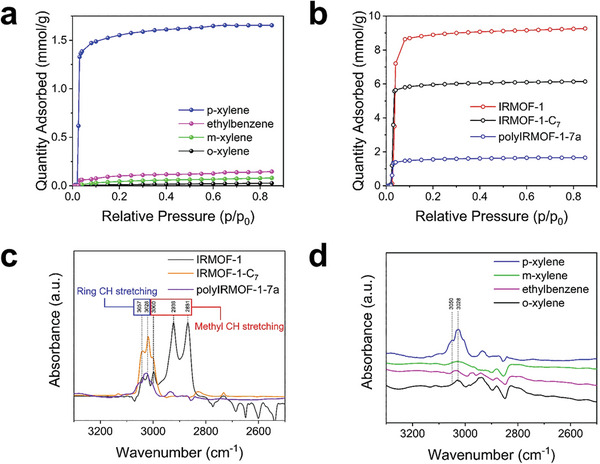
a) Unary vapor phase adsorption isotherms of xylene isomer at 298K in polyIRMOF‐1‐7a. b) Unary vapor phase adsorption isotherms of p‐xylene at 298K in IRMOF‐1, IRMOF‐1‐C_7_, and polyIRMOF‐1‐7a. c) In situ diffuse reflectance infrared spectroscopy (DRIFTS) of IRMOF‐1 derivatives with the nitrogen flow consisting of 5% of p‐xylene and d) in situ DRIFTS of polyIRMOF‐1‐7a with the nitrogen flow consisting of 5% of each xylene isomer. Each xylene isomer was adsorbed onto the MOFs (IRMOF‐1, IRMOF‐1‐C_7_, and polyIRMOF‐1‐7a) at 323K for 0.5 h to prevent condensation.

Unary gravimetric vapor sorption experiments at 298 K were conducted for the MOF crystals prepared in this work for various benzene‐derivatives (B; benzene, T; toluene, EB; ethylbenzene, p‐X; para‐xylene, o‐X; ortho‐xylene, m‐X; meta‐xylene). As expected, non‐selective adsorptions of xylene isomers were observed for IRMOF‐1 and IRMOF‐1‐C_7_, where both materials possessed larger pore sizes than polyIRMOF‐1‐7a. (**Figure** **2**b; Figure S13, Supporting Information) Notably, polyIRMOF‐1‐7a showed exceptionally selective p‐X uptake at 0.85 activity (1.65 mmol g^−1^) compared to other isomers (0.03, 0.08, and 0.14 mmol g^−1^ for o‐X, m‐X, and EB respectively, Figure 2a). The ideal selectivity for p‐X vapor was 11, 21, and 55 toward EB, m‐X, and o‐X, respectively, showing size‐dependent molecular sieving of polyIRMOF‐1‐7a. In the case of other polyMOFs crystallized with shorter (C_5_ and C_6_) alkyl bridging chains (polyIRMOF‐1‐5a and polyIRMOF‐1‐6a), non‐selective sorption among xylene was observed with much smaller adsorption capacity than polyIRMOF‐1‐7a (Figure S14, Supporting Information). The poor adsorption performance of the polyIRMOF‐1‐5a, which has a micropore similar to polyIRMOF‐1‐7a, will be further discussed in the upcoming stability section. On the other hand, the highly selective adsorption of p‐X in polyIRMOF‐1‐7a could be attributed to the optimal pore dimension (5.8 Å) with pore‐traversing alkyl chain, which can exclusively sieve out the smallest p‐X (5.8 Å) over m‐X (6.8 Å), o‐X (6.8 Å) and EB (6.0 Å), via enthalpic contribution (i.e., size selectivity). Up to 318K, the opening pressure of p‐xylene within polyIRMOF‐1‐7a remained unchanged, suggesting the pore rigidity of polyIRMOF‐1‐7a (Figure S15, Supporting Information).^[^ ^38^ ^]^ For an accurate comparison, the adsorption of benzene (5.8 Å) and toluene (5.8 Å) vapors were also tested for polyIRMOF‐1‐7a since those molecules have similar kinetic diameters to p‐xylene (Figure S16, Supporting Information). Toluene showed a similar saturated uptake amount (1.99 mmol g^−1^) with p‐xylene, while benzene showed the highest uptake (3.5 mmol g^−1^). The result of unary vapor adsorption suggested the precise size‐based cut‐off at the range of C_8_ aromatics in polyIRMOF‐1‐7a.

The in situ diffuse reflectance infrared spectroscopy (in situ DRIFTS) was used to understand the molecular arrangement of the adsorbed xylene species at the surface of IRMOF‐1 derivatives (Supplementary Information, Materials and methods). As a reference, each variant of xylene was first adsorbed onto the surface of Al_2_O_3_ to identify the location of the distinctive absorption peaks (Figure S17, Supporting Information). Consistent with the simulated IR spectrum, peaks at 3049 and 3020 cm^−1^ originated from the C‐H stretch in the benzene ring, and others (3000, 2928, and 2874 cm^−1^) were from methyl groups in the reference Al_2_O_3_ case (Figures S17 and S18, Supporting Information).^[^ ^39^ ^]^ The same C‐H stretching peaks of p‐X were observed in all IRMOF‐1 derivatives (Figure 2c), suggesting the physical adsorption of p‐X on the surface of IRMOF‐1 derivatives was similar to the reference case. Furthermore, the magnitude of the IR peak intensities was matched with the adsorbed p‐X amount at equilibrium shown in Figure 2b. Interestingly, restricted methyl C‐H stretching mode of xylene isomers was observed for the MOFs created with any alkyl bridging ligands, IRMOF‐1‐C_7,_ and polyIRMOF‐1‐7a. The negligible C‐H stretching peak of p‐xylene indicates the strong interaction between the methyl group and the surface. It is hypothesized that the molecular orientation of the p‐X was hindered due to the presence of an alkyl chain across the pore window (i.e., ‘configuration 2′ in IRMOF‐1‐C_7_) or the presence of a traversing alkyl chain within the pore (i.e., ‘configuration 3′ in polyIRMOF‐1‐7a). A similar phenomenon was also reported with different MOFs (e.g., Christian‐Albrechts‐University‐1, CAU‐1), which had a pore size similar to that of xylene isomers.^[^ ^40^ ^]^ In situ DRIFTS further confirmed the selective adsorption p‐X over other variants, probed by the absence of IR absorption peak of other xylene isomers in polyIRMOF‐1‐7a (Figure 2d).

**Figure 3:**
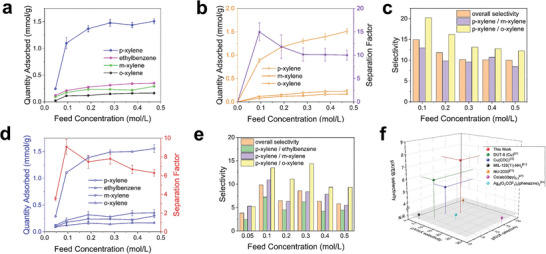
a) Unary liquid batch adsorption isotherms of xylene isomer at 298K in polyIRMOF‐1‐7a. b) Ternary mixture liquid batch adsorption isotherm at 298K for polyIRMOF‐1‐7a. c) Selectivity chart at various feed concentration of ternary mixture experiment. d) Quaternary mixture liquid batch adsorption isotherm at 298K for polyIRMOF‐1‐7a. e) Selectivity chart at various feed concentration of quaternary mixture experiment and f) p‐xylene selectivity toward m‐xylene, o‐xylene, and ethylbenzene of state‐of‐art p‐xylene selective adsorbents at 298K in liquid phase. For the adsorbents with unknown p‐xylene selectivity toward ethylbenzene, N.R. (Not Reported) value was assigned and expressed by gray color shaded area.

Unary liquid phase adsorption experiments were conducted to validate the preferential uptake of p‐X in the liquid phase. For the preparation of the feed solution, each xylene isomer was diluted by 1,3,5‐triisopropylbenzene (TIPB, 8.5 Å) as a non‐adsorbing solvent for 0.05–0.5 m concentration and n‐undecane was used as an internal standard for analysis. Activated polyIRMOF‐1‐7a (60 mg) was soaked in each feed solution at room temperature. The adsorption amount was quantified by measuring supernatant concentrations before and after the adsorption process using gas chromatography (Supplementary Information, Materials and Methods). Adsorption amount at room temperature for 0.5 m feed solution was 1.51 mmol g^−1^ for p‐X and 0.35 mmol g^−1^, 0.29 mmol g^−1^, and 0.16 mmol g^−1^ for EB, m‐X and o‐X respectively (**Figure** **3**a). The ideal selectivity for the liquid p‐X was 4.3, 5.2, and 9.4 toward EB, m‐X, and o‐X, respectively. Preferential p‐X adsorption was also reproduced in unary liquid adsorption, matched with the result of unary vapor adsorption. Regardless of the adsorbate phase, selective adsorption of p‐X over other isomers was observed in polyIRMOF‐1‐7a, illustrating its potential as an efficient adsorbent for separating xylene mixtures (Table S4, Supporting Information).

### 2.3 Ternary and Quaternary Xylene Uptake Performance in Liquid Phase

Based on the unary xylene adsorption results, we investigated the capability of polyIRMOF‐1‐7a for selective recognition of p‐X in liquid xylene mixtures. Activated polyIRMOF‐1‐7a (60 mg) was soaked in the equimolar mixture of p‐X, m‐X, and o‐X at 298K with feed concentration from 0.1 to 0.5 m. To avoid the interference in gas chromatography (GC) peaks for p‐X and m‐X in similar positions (Figure S20, Supporting Information), which might lead to errors in selectivity calculation, the adsorption amount of each isomer was quantified by ^1^H solution NMR for enhanced accuracy. We measured the difference in supernatant concentrations before and after the adsorption process. Following the adsorption process, the intensity of the characteristic peak for p‐X (2.23 ppm, denoted as peak **2**) exhibited an exclusive reduction compared to the peak intensity observed in the initial feed solution across all feed concentrations (Figure S19, Supporting Information). The overall separation factor of p‐X over other isomers (α_p/mo_) was ≈15 at 0.1 m, and the equilibrium adsorption amount at 0.5 m was 1.51 mmol g^−1^. Even in the ternary mixture, polyIRMOF‐1‐7a exhibited a pronounced p‐X selectivity, identical to the result of unary liquid batch adsorption (Figure 3b). This indicates the cross‐effects in multicomponent condition was insignificant during the adsorption process. The p‐X/o‐X selectivity was the highest in most feed concentrations, even showing a selectivity of 20 at 0.1 m concentration (Figure 3c).

Since the composition of xylene mixtures used in a conventional SMB process included a minor component of ethylbenzene,^[^ ^25^ ^]^ we additionally performed a batch adsorption experiment with equimolar quaternary xylene mixture of p‐X/m‐X/o‐X/EB. The same experimental process proceeded for the quaternary mixture, except for the quantification method using gas chromatography (GC), since other xylenes in the NMR spectrum blinded the characteristic peak of EB. The reduction in peak intensity associated with p‐X in supernatant solution was evident compared to the peak intensity observed in the initial feed solution across all concentrations (Figure S20, Supporting Information). PolyIRMOF‐1‐7a showed an exceptionally selective p‐X uptake of 1.56 mmol g^−1^ compared to other isomers in quaternary mixtures. The overall selectivity of p‐X over different isomers (α_p/ome_) was 9 at 0.1 m and gradually decreased as feed concentration increased (Figure 3d). For binary selectivity obtained from the quaternary experiment, p‐X/o‐X selectivity was up to 13.5, and p‐X/m‐X was 10.9 at 0.1 m feed concentration (Figure 3e). Even for the quaternary mixtures, precise discrimination of p‐X among other isomers was also feasible for polyIRMOF‐1‐7a, showing the existence of favorable microscopic space provided by the traversing alkyl chain in the pores. Compared to other reported MOFs for xylene isomer separation at 298K, polyIRMOF‐1‐7a showed outstanding p‐X selectivity toward both m‐X and o‐X (Figure 3f). Furthermore, polyIRMOF‐1‐7a can even discriminate p‐X over EB in the quaternary mixture, which is rarely achieved or reported due to the very similar kinetic diameter between those (Table S4, Supporting Information). For all the adsorption experimental campaigns, including vapor and multicomponent liquid mixtures, polyIRMOF‐1‐7a exhibited consistent adsorption and outstanding separation performances for the purification of para‐xylene.

### 2.4 Stability of polyIRMOF‐1‐7a

Many porous adsorbents reported for adsorptive separation suffered from poor stabilities, particularly MOFs with open metal sites.^[^ ^41^ ^]^ In contrast, polyIRMOF‐1‐7a was highly resistant to heat and air, as demonstrated by the synchrotron supramolecular crystallography (SMC) and powder X‐ray diffraction (PXRD) (Figures S22 and S23, Supporting Information). The crystallinity of polyIRMOF‐1‐7a was maintained up to 450 K in ambient air conditions and preserved even after the xylene adsorption experiments for both vapor and liquid phases. The porosity of polyIRMOF‐1‐7a was preserved following the unary vapor phase adsorption of p‐xylene (Figure S24, Supporting Information). Furthermore, p‐xylene uptake amount of polyIRMOF‐1‐7a was preserved upon three cycles in unary vapor phase adsorption experiment (Figure S25, Supporting Information). The remarkable resistance of polyIRMOF‐1‐7a was attributed to the increased hydrophobicity by the inserted alkyl chains of polymeric ligands.^[^ ^32^ ^]^ Hydrophobicity is one of the essential features in discussing the stability of MOFs, and the hydrophobicity of polyIRMOF‐1‐xa synthesized in this study was further studied by measuring the water contact angle. PolyIRMOF‐1‐7a was observed to be hydrophobic with an initial water contact angle of ≈92°, and polyIRMOF‐1‐5a was hydrophilic (Figure S26, Supporting Information). The hydrophobic nature of polyIRMOF‐1‐7a is exclusively attributed to the hydrophobic nature of the longer alkyl chain in pbdc‐7a, distinct from pbdc‐5a and −6a (Figure S27, Supporting Information).^[^ ^32^ ^]^ Enhanced hydrophobicity of polyIRMOF‐1‐7a makes it more resistant to the activation and adsorption process than polyIRMOF‐1‐5a, which quickly loses its crystallinity after the xylene adsorption experiment (Figure S28, Supporting Information).

To further investigate the impact of alkyl chain length on the stability of the polyIRMOF‐1‐xa structure, we analyzed the degree of torsion induced in the benzene ring of the bdc ligands using computational methods. The extent of torsion applied to the ligands is directly related to the degree of stress experienced by the structure, which can serve as a measure of physical and mechanical stability. The torsion acting on the benzene ring of the bdc ligand was calculated for alkyl chains of different lengths (x = 5 to 7) connecting two vertically aligned bdc ligands (‘configuration 1′, Figure S4, Supporting Information). Torsion calculation was carried out in ‘configuration 1′ for fair comparison, as the precedent research reported that the alkyl chains with x = 5 and 6 were not long enough to be stably inserted into other configurations (2 to 4).^[^ ^34^ ^]^ As shown in Figure S29 (Supporting Information), it was found that alkyl chains of length x = 5 and 6 induced more torsion on the benzene ring than x = 7. This would be attributed to the fact that alkyl chains of x = 5 and 6 are not long enough to link the two bdc ligands without stressing the entire structure. The relatively low crystallinity observed in polyIRMOF‐1‐5a and polyIRMOF‐1‐6a may be due to the high torsion, as the stability of the structure decreases with increasing stress acting on the structure. Moreover, in addition to the restrained torsion exerted on the ligands, pronounced hydrophobicity contributes to the superior structural stability of polyIRMOF‐1‐7a, suggesting its potential as a practical adsorbent applicable to the separation of liquid hydrocarbons.

## 3 Conclusion

A hybrid porous material between polymer and MOF was introduced more than a decade ago. PolyMOFs were repeatedly synthesized in the literature with different metal sources and polymeric ligands, however, the application field of polyMOFs was somewhat unclear. DFT calculations conducted in this work demonstrated that the length and molecular arrangement of the bridging alkyl chain would substantially influence the constraints and ultimately govern the separation capabilities within the polyMOFs. Combining the experiments with computational studies, we suggest that the structure of polyIRMOF‐1‐7a would involve a significant portion of the alkyl chain connecting the bdc ligands in a pore traversing position (‘configuration 3′). The penetration of the alkyl chains in the pores significantly decreases the pore volume and size compared to its parent IRMOF‐1. This leads to the optimal pore dimension for the selective adsorption of p‐xylene over other benzene derivatives. In situ DRIFTS IR spectrum also supports the different molecular interactions for p‐xylene when the bridging alkyl chains are placed within the pore. Consistent with unary vapor and liquid adsorptions, polyIRMOF‐1‐7a showed exceptional p‐xylene selectivity (α_pX/OM_ = 15 and α_pX/OME_ = 9) even for both ternary and quaternary liquid mixture batch adsorptions, which are compared to state‐of‐the‐art MOF based adsorbents (Figure 3f). Incorporating amorphous polymers into crystalline MOFs results in highly stable and highly selective porous materials that can act as selective molecular sieves. The structural ambiguity concerning polyIRMOF‐1‐7a was not entirely resolved owing to the lack of a direct observation method for alkyl chains in polyIRMOF‐1‐7a. However, elucidating the polymer chain configuration within polyIRMOF‐1‐7a can be achieved with greater precision by examining the in situ nucleation and growth process of polyIRMOF‐1‐7a through sophisticated analytical techniques and in‐depth simulation tools.

Explaining the inherent chemical diversity within polymeric ligands is a significant advantage, enabling further modification of polyMOFs to align with specific purposes.^[^ ^42^ ^]^ With the principle of isoreticular chemistry, the pore size of polyMOFs can also be expanded through the linear extension of organic linker groups.^[^ ^43^ ^]^ In other words, the pore dimension of polyMOF can be flexibly expanded or reduced by regulating both the ligand's inherent length and the length of the alkyl chain bridging the ligand units. It offers the potential to modify the pore dimension of diverse MOFs, with the anticipation of their application in a multitude of adsorptive separation processes. Not only for internal pore dimension, morphologies and sizes of polyMOF crystals can be controlled by hybridization of the block copolymers with MOFs.^[^ ^44^ ^]^ It can lead to the development of membranes that integrate MOFs and polymers at the molecular level, thereby maximizing the expression of their respective optimal characteristics.^[^ ^45^ ^]^

Despite the potential applications of remarkable self‐assembly, designing a polymeric ligand capable of crystalline formation with a precise chemical reaction is undoubtedly challenging. Only a limited number of polyMOFs using polymers containing carboxylate groups have been reported since elucidating the atomic‐level structure and dynamics of polyMOF materials presents its own set of complexities. If subsequent investigations can precisely clarify the formation of polyMOF structures, it could lead to the emergence of diverse polyMOF types and an extended application of hybridization strategy to various crystalline materials, such as covalent organic frameworks (COFs) and polymer‐metal‐organic cages (polyMOCs).^[^ ^46^, ^47^ ^]^ We expect that this polymer‐to‐MOF hybridization strategy can lead to substantial improvements in the development of next‐generation adsorbents, anticipated to provide a “breakthrough” to address the constraints associated with current adsorbent materials.

## 4 Experimental Section

Detailed material synthesis, characterization data, and computational methods are listed in the Supporting Information.

## Conflict of Interest

The authors declare no conflict of interest.

## Supporting information

Supporting Information

## Data Availability

The data that support the findings of this study are available from the corresponding author upon reasonable request.
