# Supplementary material for: Unexpected Molecular Sieving of Xylene Isomer Using Tethered Ligand in Polymer‐Metal–Organic Frameworks (polyMOFs)
Source: Adv Sci (Weinh). 2024 Jul 8;11(34):2402980. doi: 10.1002/advs.202402980 (PMC11425841; doi:10.1002/advs.202402980)
Supplement: Supplementary file 1 — Supporting Information [file ADVS-11-2402980-s001.docx]

Supporting Information

**Unexpected Molecular Sieving of Xylene Isomer using Tethered Ligand in Polymer-Metal-Organic Frameworks (PolyMOFs)**

*Taehoon Hyun, Junkil Park, Jungseob So, Jihan Kim* and Dong-Yeun Koh**

T. Hyun, J. Park, J. Kim, D.-Y. Koh

Department of Chemical and Biomolecular Engineering (BK-21 Plus), Korea Advanced Institute of Science and Technology, Daejeon, 34141, the Republic of Korea

E-mail: dongyeunkoh@kaist.ac.kr, jihankim@kaist.ac.kr

Jungseob So

Environment & Sustainable Resources Research Center, Korea Research Institute of Chemical Technology, Daejeon, 34114, the Republic of Korea

**Supporting Information includes**:

Materials and Methods

Table S1 to S4

Figs. S1 to S29

References

**Materials and methods**

**Materials and general synthetic scheme**

All of used materials and solvents were purchased and used without additional purification (Sigma-aldrich, Alfa aesar, TCI). Deuterium solvents for ^1^H nuclear magnetic resonance (NMR) spectra were purchased from Cambridge Isotope Laboratories.


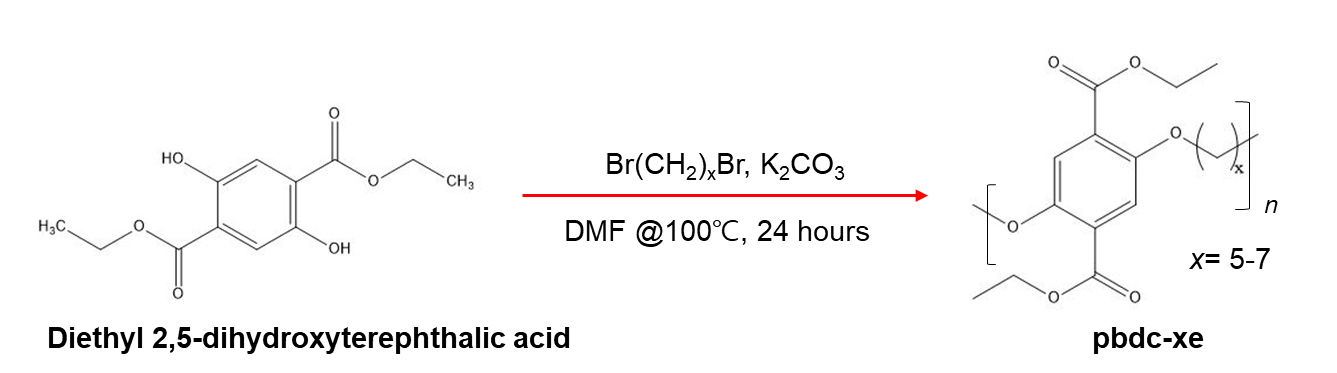


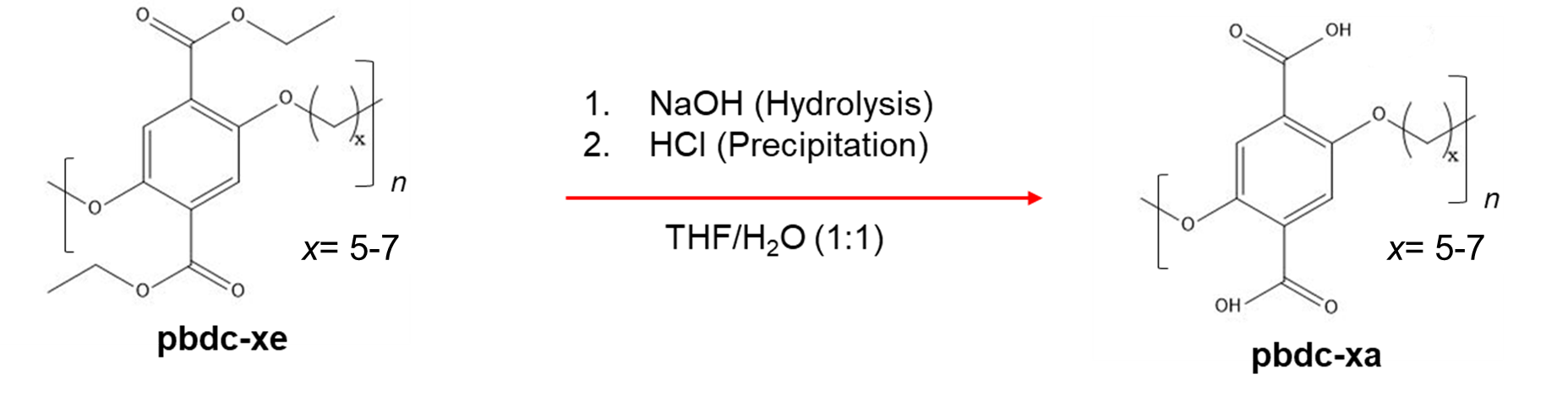


**Scheme S1**. General synthesis procedure of pbdc-xa

**Polymeric ligand pbdc-xa (x= 5 to 7) synthesis**

Diethyl 2,5-dihydroxyterephthalic acid (1.2 g, 4.7 mmol), dibromoalkane (4.75 mmol) and potassium carbonate (2.6g, 19.0 mmol) were added in 20 mL N,N-dimethylformamide (DMF). Reaction mixtures were heated at 100℃ for 24 hours with stirring. After the polymerization was completed and cooling, 50 mL of deionized water was added to suspension. Suspensions were transferred to centrifuge tube and polymers were isolated by centrifuge (6000 rpm, 10 min). Supernatant was decanted and obtained solids (**pbdc-xe**) were washed with methanol thoroughly (50 mL for 3 times) with vacuum filtration. After the vacuum filtration, solids were dried in vacuum oven at 40℃ for overnight. The yields of **pbdc-xe** was nearly 70%. For the hydrolysis of ester group in **pbdc-xe**, 1.0 g of polymers were placed in a 1:1 mixture solution (30 mL total) of water and tetrahydrofuran (THF). Then, 2.82 g of sodium hydroxide (NaOH) was added and mixture was stirred for 24 hour. The THF was eliminated through evaporation. After the THF evaporation, 4M hydrogen chloride (HCl) solution was gradually added until the pH of solution becomes ~1. Obtained solid (**pbdc-xa**) was washed thoroughly with 4M HCl solution and deionized water to remove any salts. All samples were dried in vacuum oven at 40℃ for overnight.

**
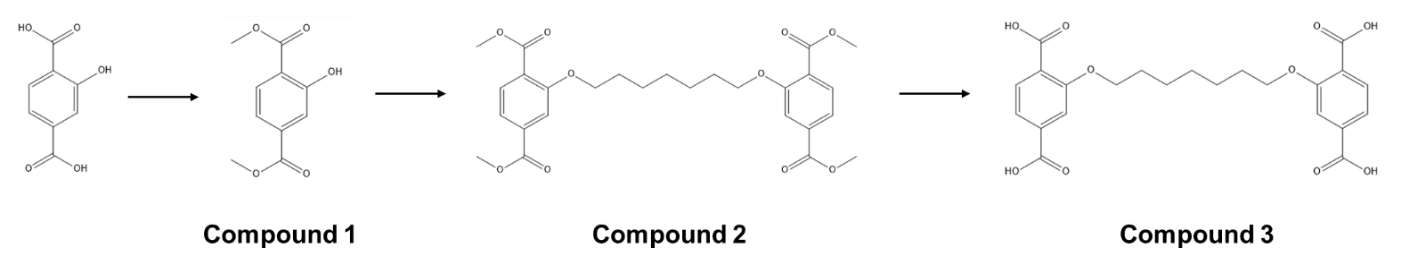
Cross-linked bdc ligand (1,7-Bis[2,5-dicarboxyphenoxy]heptane) synthesis**

Modified synthetic procedure was utilized ^[1]^. First, 2-hydroxyterephthalic acid (2.42g, 13.3 mmol) was dissolved in a 250 mL of MeOH. 5mL of concentrated sulfuric acid was added and mixture was heated with reflux overnight (~18 hours). After cooling to room temperature, solution was neutralized with saturated NaHCO_3_ solution. Precipitates were thoroughly washed with water and dried. Dried dimethyl 2-hydroxyterephthalate (Compound 1, 1.50 g, 7.1 mmol) and 1,7-dibromoheptane (0.877 g, 3.4 mmol) were dissolved in 15 mL of DMF and K_2_CO_3_ (1.88 g, 13.6 mmol) was added. Mixture was heated up to 80℃ and reacted overnight. After the cool down, suspension was filtered to eliminate undissolved K_2_CO_3_ and excess water was put into suspension and beige precipitate was isolated by vacuum filtration. Resulting solid (Compound 2, 0.7g, 1.4 mmol) was dissolved in 1:1 (v/v) THF/4% KOH (aq) solution (30 mL) and stirred during overnight at room temperature. Aqueous layer was acidified to pH~1 with 1M HCl solution. A white precipitate (Compound 3) was isolated by vacuum filtration

**^1^H Nuclear magnetic resonance (NMR) data for pbdc-xa (x=5 to 7)**

^1^H NMR spectra was obtained through Avance Neo 600 (600 MHz, Bruker) equipped with active shielded 14.1 Tesla NMR spectrometer

**Compound 1**: (600 MHz, CDCl_3_) δ = 3.92 (s, 3H; CO2CH3), 3.98 (s, 3H; CO2CH3), 7.52 (dd, 3J(H,H)=8 Hz, 4J(H,H)=1.6 Hz, 1H; ArH), 7.63 (d, 4J(H,H)=1.6 Hz, 1H; ArH), 7.90 (d, 3J(H,H)=8.4 Hz, 1H; ArH).

**Compound 2**: (600 MHz, DMSO-d_6_) δ = 1.73 (m, 2H; CH2), 1.95 (m, 4H, CH2),3.89 (s, 6H, CO2CH3), 3.93 (s, 6H, CO2CH3), 4.13 (t, 4H; CH2), 7.60(d, 4H; ArH), 7.8 (d, 2H; ArH)

**Compound 3**: (600 MHz, DMSO-d_6_) δ =7.66 (d, 8.1 Hz, 2H), 7.55 (s, 2H), 7.53 (d, 8.0 Hz, 2H), 4.08 (t, 6.0 Hz, 4H), 1.74 (m, 4H), 1.47 (m, 4H), 1.40 (m, 2H)

**pbdc-5a**: (600 MHz, DMSO-d6): δ = 7.27 (s, 2H), 3.98 (t, 4H), 1.74 (t, 4H), 1.57 (m, 2H)

**pbdc-6a**: (600 MHz, DMSO-d6): δ = 7.26 (s, 2H), 3.98 (t, 4H), 1.69 (t, 4H), 1.56-1.25 (m, 4H)

**pbdc-7a**: (600 MHz, DMSO-d6): δ = 7.25 (s, 2H), 3.97 (t, 4H), 1.70 (t, 4H), 1.51-1.18 (m, 8H)

**PolyIRMOF-1-xa synthesis**

Zinc nitrate hexahydrate, Zn(NO_3_)_2_·6H_2_O (0.60 mmol) and pbdc-xa (0.10 mmol, based on monomer repeating unit) were placed in 10mL glass vial with 2.5 mL DMF. The vial was heated at 100℃ for 24 hours. Obtained solids were washed with DMF.

**Fourier transform Infrared Spectroscopy (FT-IR)**

Attenuated total reflectance Fourier transform infrared spectra was obtained through Nicolet iS50 FT-IR spectrometer (Thermo Scientific) in transmittance mode. Spectra range was set from 400 to 4000 cm^-1^, resolution of 2 cm^-1^. Drift cell (DIFFUSIR, pike technology Inc.) was equipped for in-situ FTIR analysis.

**In-situ diffuse reflectance infrared spectroscopy (DRIFTS)**

In-situ IR experiment was conducted as follows. First, *ex-situ* vacuum degassing was performed for the samples. Each substrate (p-xylene, m-xylene, o-xylene, and ethylbenzene) in the N_2_ balance, comprising 5% of the total, was introduced into the IR cell. All lines were heated up to 373K to prevent condensation in the line. The substrates were adsorbed onto the MOFs at 323K for 0.5 hours to prevent condensation on the MOFs. Finally, the IR cell was flushed with Ar flow.

**Field Emission Scanning Electron Microscopy (FE-SEM)**

SEM image was obtained through Magellan 400 (FEI company, USA) field emission scanning electron microscopy equipped with Schottky thermal field emitter with UC (< 0.2 eV energy spread). Images were obtained at 10kV and 0.1 nA with a working distance of 5 mm using the in-lens detector (TLD).

**Powder X-ray Diffraction (PXRD) Characterization**

Powder X-ray diffraction experiments were performed on RIGAKU SmartLab diffractometer with a Cu K-α-1 anode (λ= 0.1542 nm). The diffraction patterns were collected over the range of 3 to 50° with a step size of 0.01° per step.

**Transmission Electron Microscope (TEM)**

Transmission Electron Microscope (TEM) images were obtained through Cryo-Field Emission TEM (Glacios, ThermoFischer), under low dose conditions and at liquid nitrogen temperature with cooling holder. Accelerating voltage was 200 kV with the minimum resolution of 0.23 nm and cryo-tomography and STEM detector was equipped.

**Thermogravimetric Analysis (TGA)**

Thermogravimetric analysis using TG209 F1 Libra analyzer (NETZSCH group, Germany) operates in the temperature range of 30 °C to 800 °C with heating rate of 5.0 K/min and air flow rate of 20 ml/min.

**Elemental Analysis (EA)**

Elemental components (C, H, and N atoms) were analyzed by Flash 2000 series (Thermo Scientific, United States). Compounds were exposed to dynamic flash combustion oxidation and pass through the chromatography column. A thermal conductivity detector analyzed the content of components. The oxygen component was continuously analyzed by the Flash 2000 series (Thermo Scientific, United States) with the same principle

**Supramolecular Crystallography**

Diffraction data was collected with synchrotron radiation (wavelength = 0.9Å) on a Rayonix MX225HS CCD detector at Supramolecular Crystallography Beamline (2D-SMC Beam Line) in the Pohang Accelerator Laboratory, Korea)

**Gas Adsorption Measurements**

Nitrogen adsorption isotherms at 77K were obtained using a volumetric physisorption apparatus (Micromeritics ASAP 2020). Prior to the gas sorption, the samples were washed with fresh DMF (24 hours) and immersed in dichloromethane (24 hours, fresh dichloromethane was supplied in every 8 hours), followed by immersion in hexane (24 hours, fresh hexane was supplied in every 8 hours). The sample was filtered then subsequently transferred into the sample tube. The degassing protocol followed two-step activation of vacuum phase with 50 °C and a subsequent heating phase with 75°C with full vacuum for 12 hours.

**Organic vapor sorption experiment**

The gravimetric vapor sorption of benzene, toluene, ethylbenzene, p-xylene, m-xylene and o-xylene was measured using a VTI-SA^+^ (TA Instruments) with relative pressure range from 0 to 0.85. Before the loading of polyIRMOF-1-5a and polyIRMOF-1-7a, each sample was preliminary activated at 75°C with vacuum for 12 hours. Samples were prepared on the quartz sample pan of apparatus, and additionally activated at 100°C for 2 hours under the nitrogen flow in the instrument before sorption test. The ideal selectivity value in the unary vapor adsorption experiment was calculated by;

$$\alpha_{p-i}=\frac{q_{p}}{q_{i}}$$

$$\alpha_{p-i}:ideal selectivity, q_{p} :p-xylene uptake amount at 1 bar (mmol g^{-1})$$

$$q_{i} :uptake amount at 1 bar \left( \mathrm{mmol}g^{-1} \right), i=ethylbenzene, m- and o-xylene$$

**Liquid batch adsorption experiment**

Before the batch experiment, each sample was activated at 75°C with vacuum for 12 hours. Xylene isomers were diluted by 1,3,5-Triisopropyl benzene (TIPB) to designed feed concentration (0.05M to 0.5M) and n-undecane was used as internal standard, which was non-adsorbing species with large molecular size. In each vial, 60 mg of activated samples were soaked in 2 mL of dilute solution with unary, ternary and quaternary xylene components. Before the measurement, solution was isolated by using a PTFE syringe filter (PTFE, hydrophobic, 0.2 μm). Concentration of xylene isomer was measured through gas chromatography (GC) or nuclear magnetic resonance (NMR). Aliquots were taken from the batch vial to calculate the adsorption amount of each isomer. For both ternary and quaternary mixtures, overlapping peaks of m-xylene and p-xylene in NMR spectra and ethylbenzene, p-xylene and m-xylene in GC data were deconvoluted by generalized Gaussian shape fitting. The uptake amount was calculated using the following equation.

$$Uptake= \frac{C_{i}-C_{f}}{m}$$

Where *C_i_* is the initial amount of each isomer in the stock solution (mmol), *C_f_* is the amount of each isomer after each aliquot (mmol), and *m* is the activated sample mass (g).

**Separation factor calculation**

The separation factor (α_ij_) was calculated based upon following equation.

$$\alpha_{ij}=\frac{q_{i}/q_{j}}{C_{i}/C_{j}}$$

In above equation, q_i_ and q_j_ are the uptake amount of component i and j, and C_i_ and C_j_ are the equilibrium concentration (mol/L) of components i and j. Overall p-xylene selectivity in ternary mixture adsorption (α­_pX/OM_) was calculated using the below equation.

$$\alpha_{pX/OM}=\frac{q_{pX}/(q_{oX}+q_{mX})}{C_{pX}/(C_{oX}+C_{mX})}$$

For the quaternary mixture adsorption, overall selectivity was calculated following equation.

$$\alpha_{pX/OME}=\frac{q_{pX}/(q_{oX}+q_{mX}+q_{EB})}{C_{pX}/(C_{oX}+C_{mX}+C_{EB})}$$

**Computational Methods**

Geometry optimization was performed using the density functional theory (DFT) method implemented in the Vienna ab initio software package (VASP)^[2]^. The project augmented wave method was employed with a kinetic energy cutoff of 520 eV. The generalized gradient approximation (GGA) using the Perdew-Burke-Ernzerhof (PBE) exchange-correlation functional was imposed with the dispersion correction using the DFT-D3 method with Becke-Jonson damping^[3,4]^. The calculations employed Γ-centered k-point grids with spacing less than 0.3 Å^–1^. During the structure optimization, a convergence criterion of 0.02 eV/Å was applied for the ionic force and 10^-4^ eV for the electronic energy. In the case of the structures where all bdc linkers converted to polymeric chain, the Forcite module of Material Studio was used for the geometry optimization due to the computational cost. During the optimization process, the interaction between the atoms was modeled using Universal Force Field (UFF) with a van der Waals cutoff radius of 12.5 Å ^[5]^. The smart minimizer algorithm was used with the moderate accuracy option. In addition to the position of the atoms, the shape of the cell was also relaxed during the optimization process. The partial charges of atoms were assigned using charge equilibration methods (Qeq) ^[6]^. Zeo++ software was used for structural analysis including pore volume, and pore size distribution calculation ^[7]^.


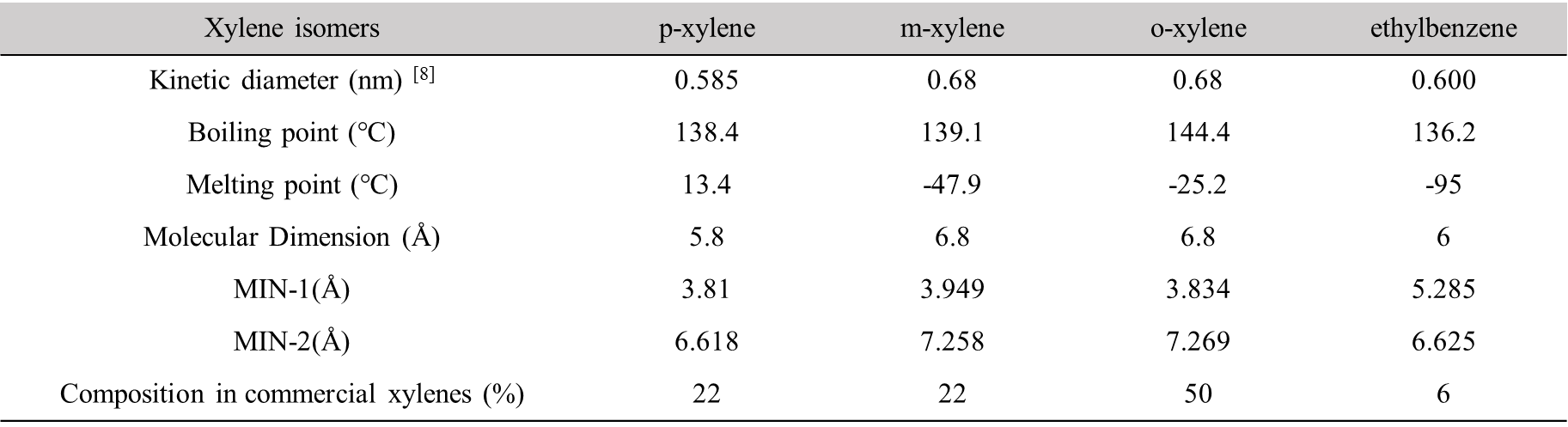


**Table S1**. Summary of physical properties of xylene isomers ^[8]^


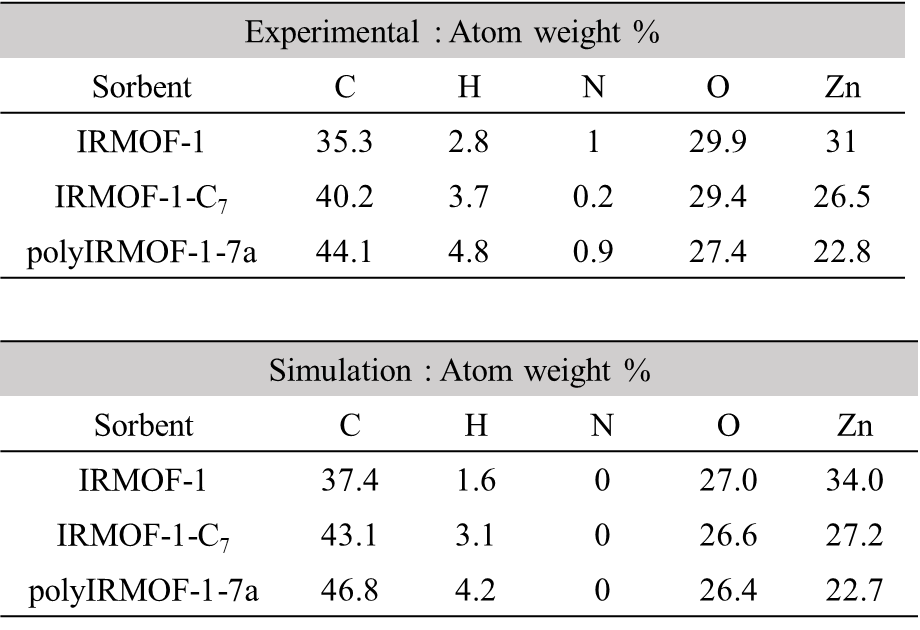


**Table S2**. Atomic weight percent of each adsorbent based on elemental analysis (top) and atomic weight percent of each adsorbent derived from simulated structure model (bottom). For simulated structure, calculation of atomic weight percent of polyIRMOF-1-7a was based on the structure model in **Figure S5** **(b)** and **(c)**. Simulated atomic weight percent was identical for each configuration in **Figure S4**.


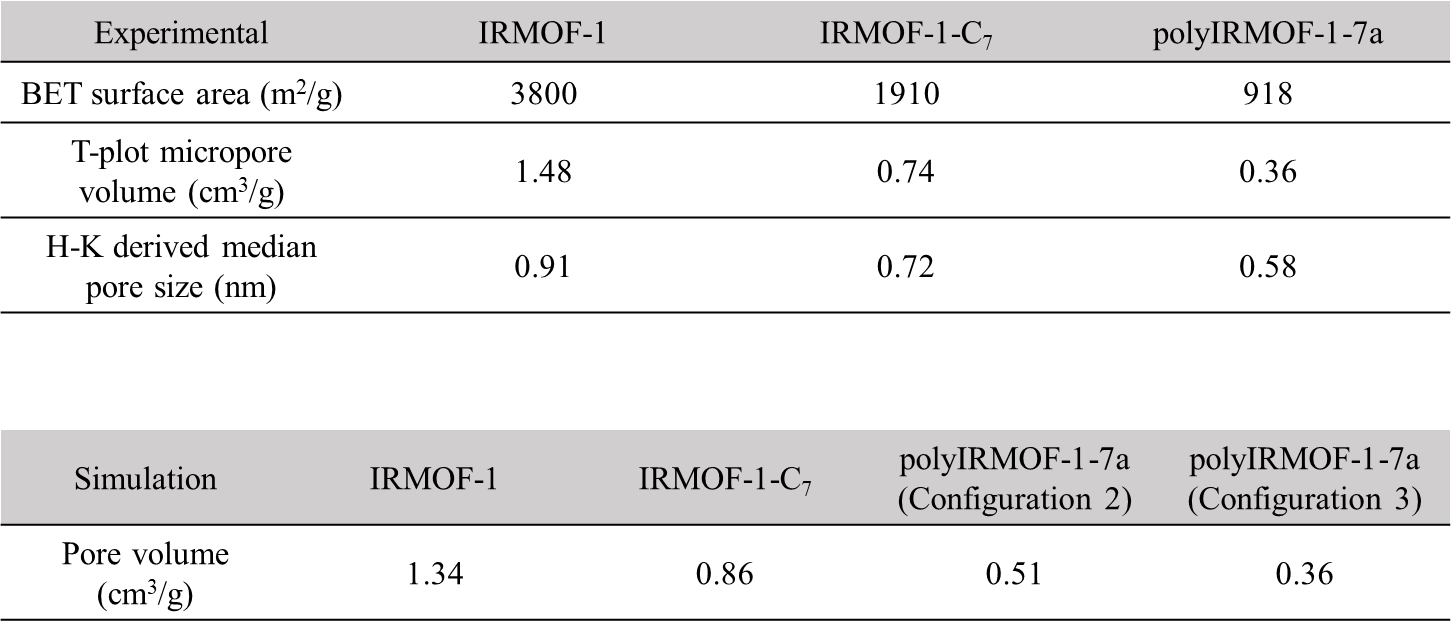


**Table S3**. 77K nitrogen physisorption derived values of BET surface area, T-plot micropore volumes and Horvath-Kawazoe (H-K) method derived median pore sizes of IRMOF-1, IRMOF-1-C_7_ and polyIRMOF-1-7a (top) and computationally calculated pore volumes (bottom). For the simulated pore volume, probe with radius of 1.86Å was used for calculation.


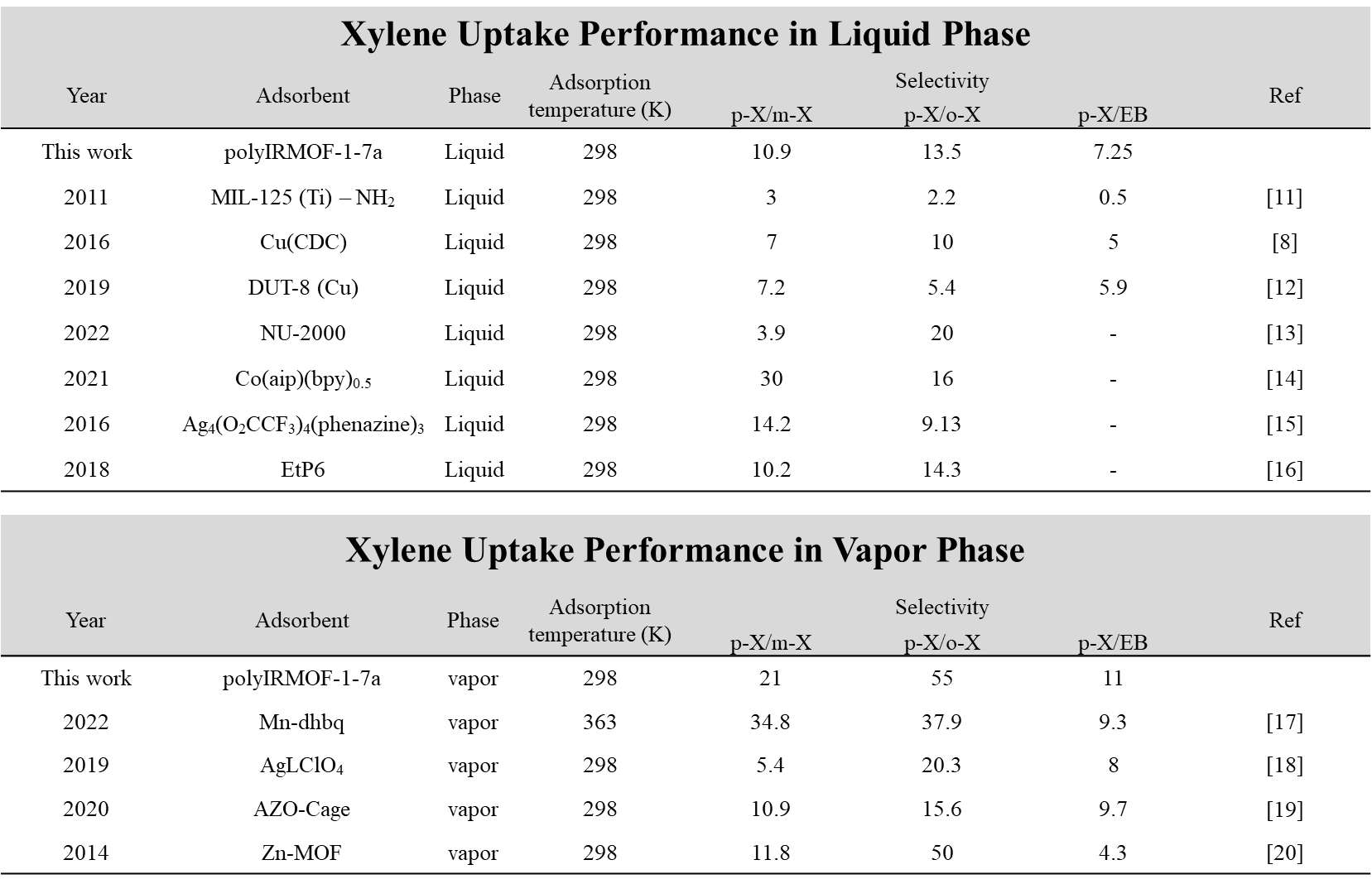


**Table S4**. Xylene uptake performance comparison with reported p-xylene selective adsorbents.


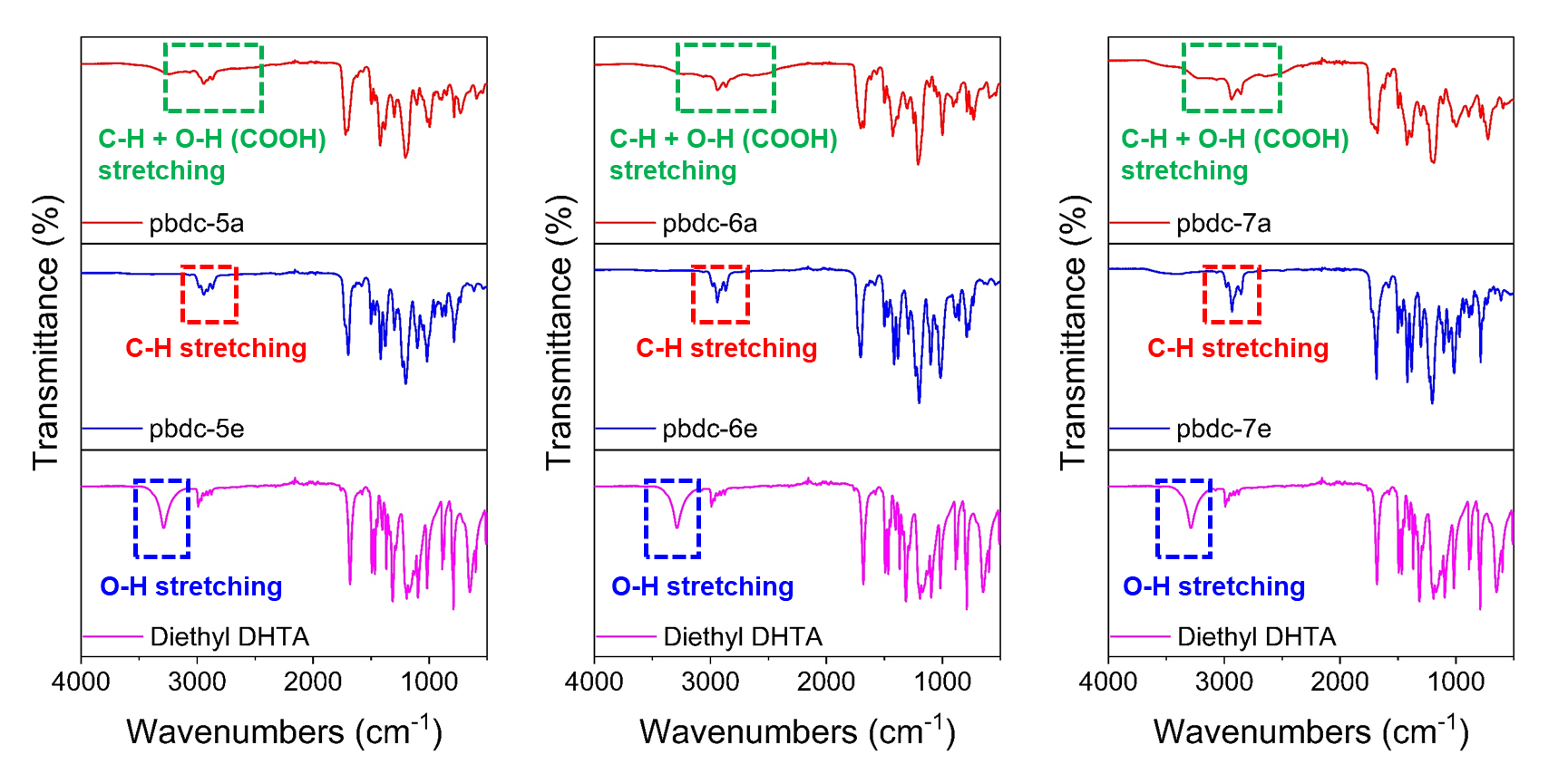


**Figure S1**. Fourier transform infrared (FTIR) spectroscopy of diethyl-dihydroxyterephthalic acid (diethyl DHTA), pbdc-xe and pbdc-xa (x=5 to 7).


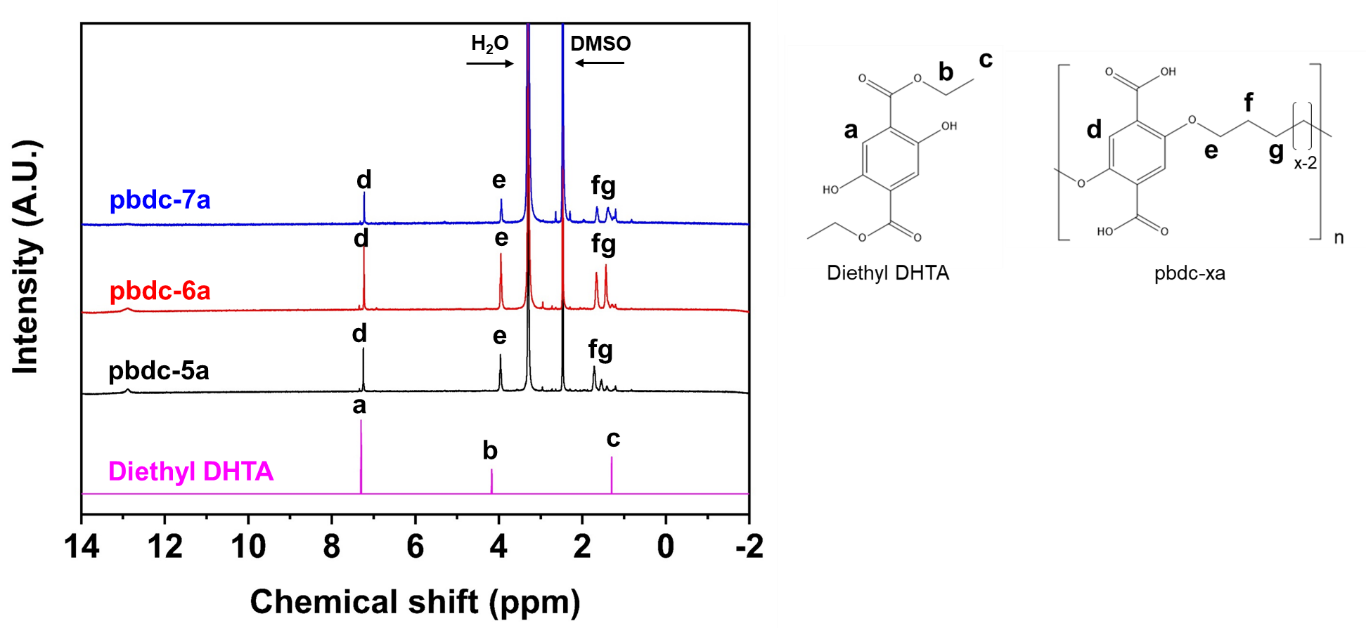


**Figure S2**. ^1^H solution NMR of pbdc-7a (blue line), 6a (red line), 5a (black line) and diethyl dihydroxyterephthalic acid (magenta line).


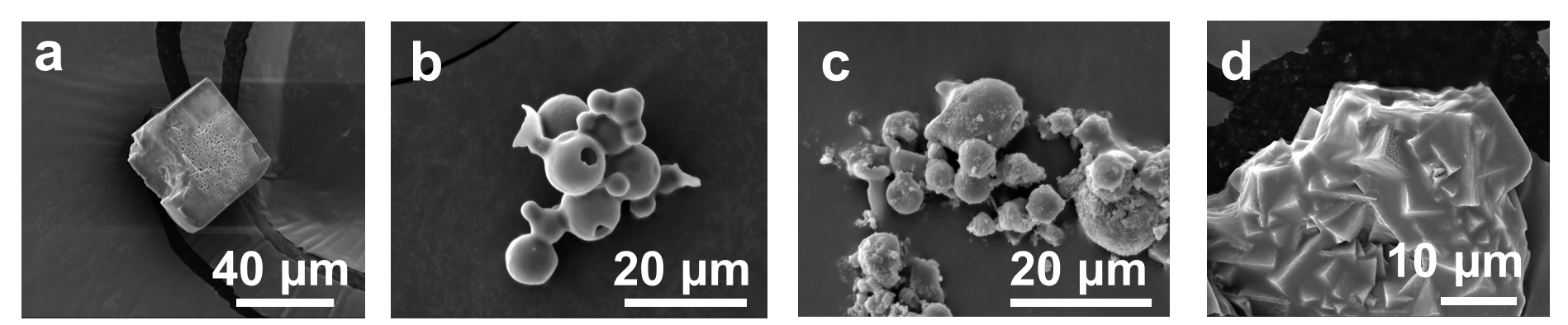


**Figure S3.** Scanning electron microscopy (SEM) image of a) IRMOF-1, b) polyIRMOF-1-5a, c) polyIRMOF-1-6a and d) polyIRMOF-1-7a.


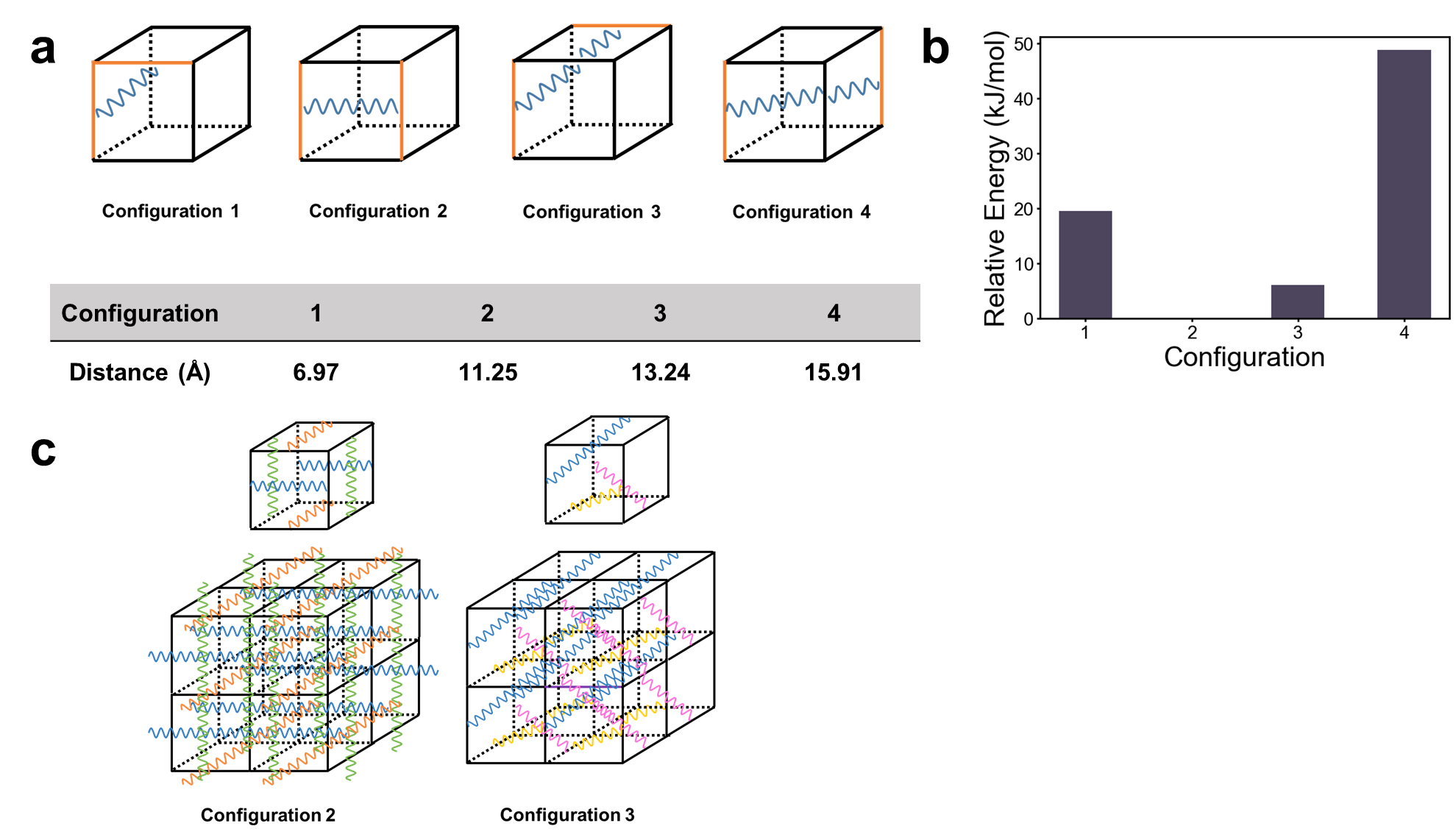


**Figure S4**. a) The possible configurations for alkyl chain insertion within polyIRMOF-1-xa, along with their corresponding distances. For simple expression, only one possible linkage was depicted for each configuration. b) Relative energy of polyIRMOF-1-7a for each configuration and c) Extended unit cells of configuration 2 and 3. Coplanar alkyl chains (linkages) were represented with identical color


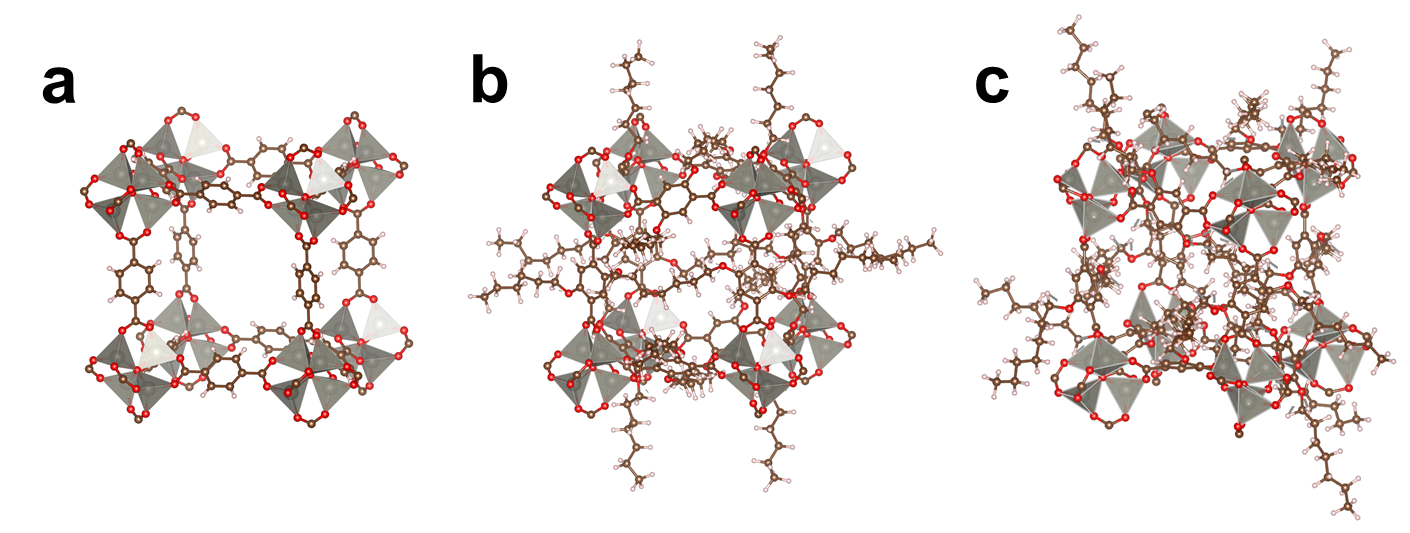


**Figure S5.** Molecular configuration of a) IRMOF-1, b) configuration 2 100% linked polyIRMOF-1-7a, and c) configuration 3 100% linked polyIRMOF-1-7a.


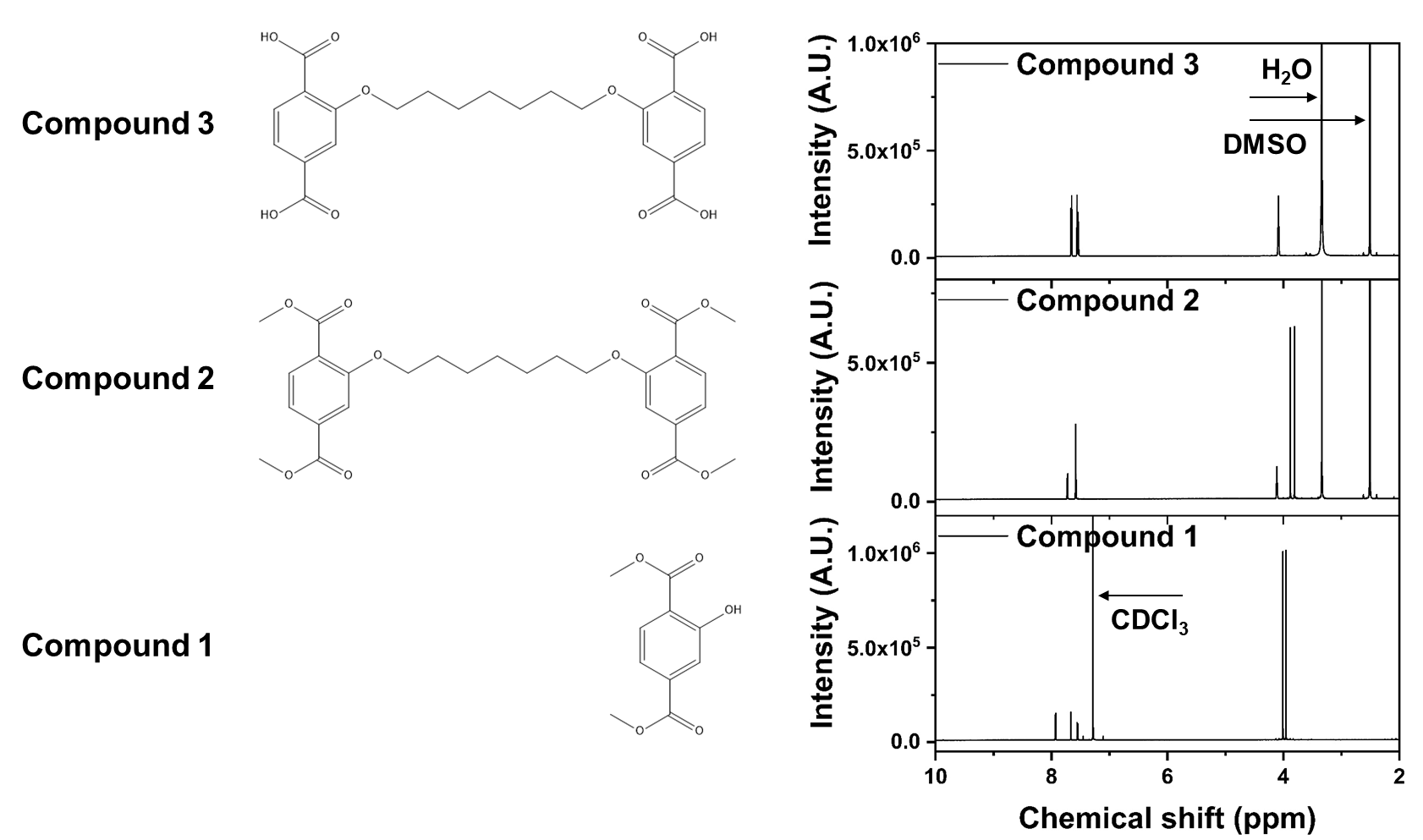


**Figure S6**. ^1^H solution NMR of compound 1 (bottom), compound 2 (middle) and compound 3 (top). Peak information was listed on material section in Supplementary Information


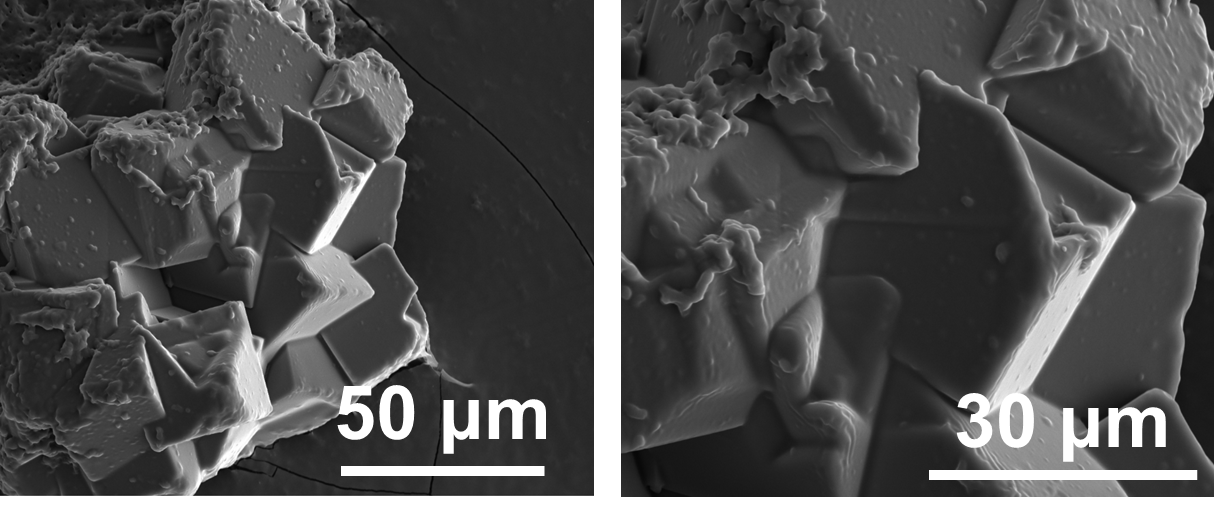


**Figure S7**. Scanning electron microscopy (SEM) image of IRMOF-1-C_7_


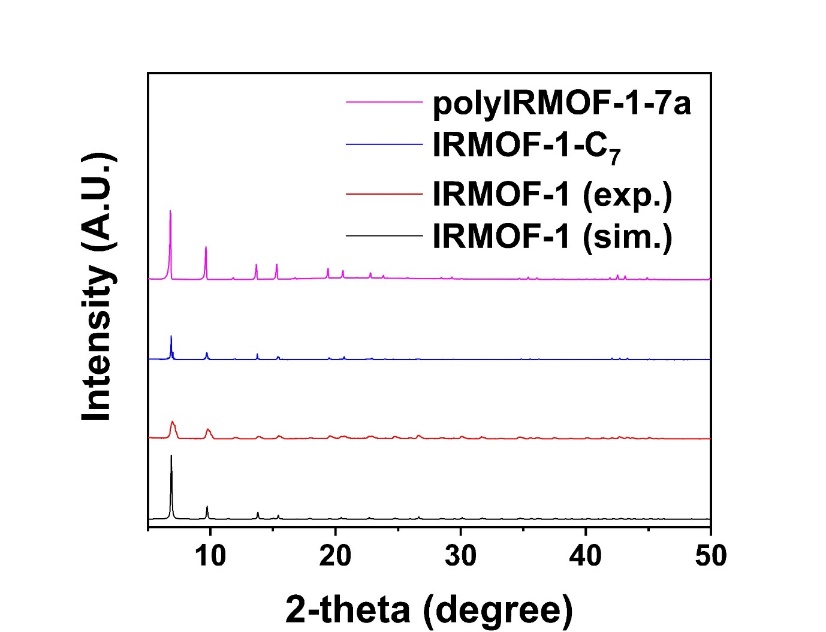


**Figure S8**. Powder x-ray diffraction data of IRMOF-1, IRMOF-1-C_7_ and polyIRMOF-1-7a


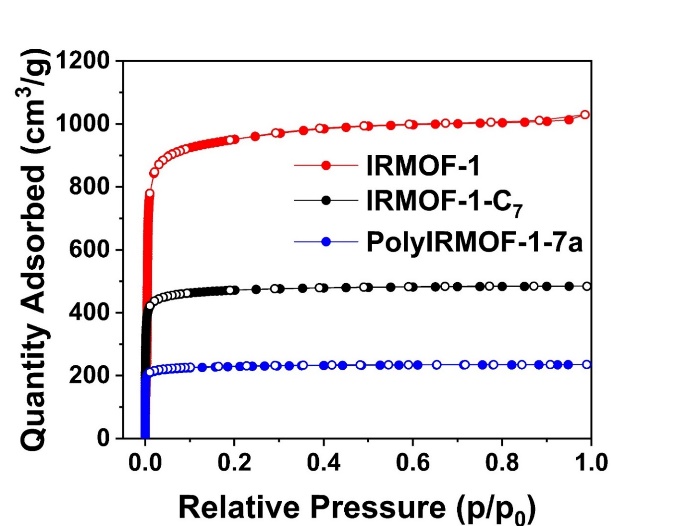


**Figure S9**. 77K nitrogen isotherms of IRMOF-1 (red), IRMOF-1-C_7_ (black) and polyIRMOF-1-7a (blue).


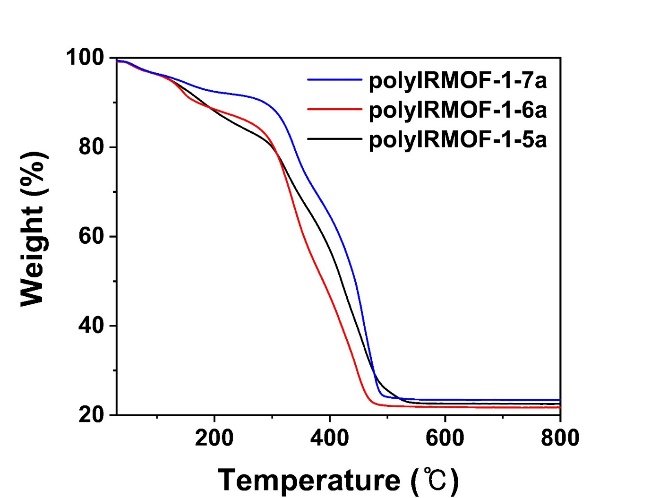


**Figure S10**. Thermogravimetric analysis (TGA) of polyIRMOF-1-7a, polyIRMOF-1-6a and polyIRMOF-1-5a


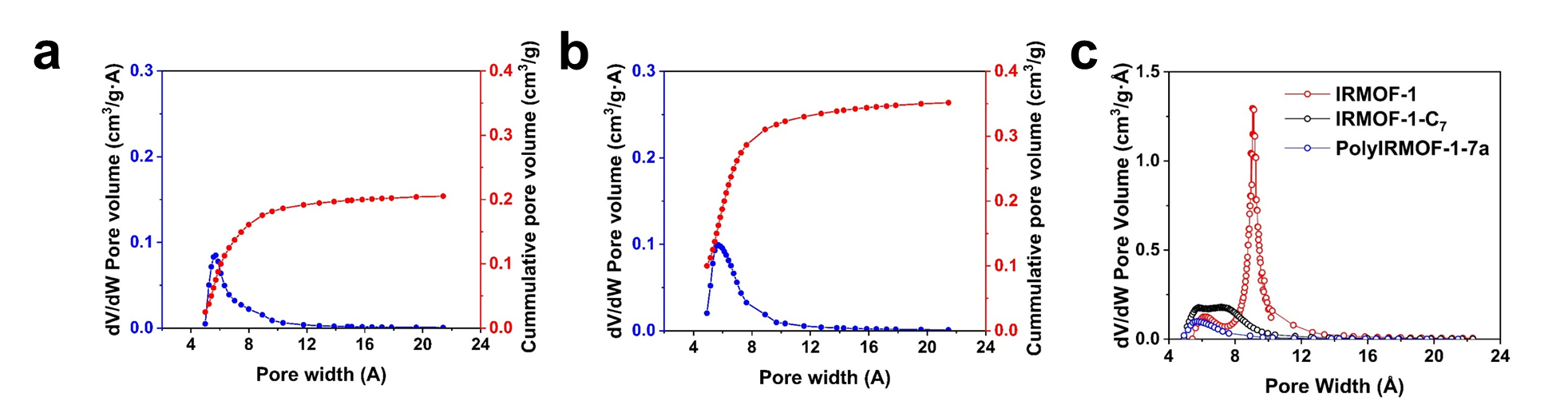


**Figure S11**. Pore size distribution of a) polyIRMOF-1-5a, b) polyIRMOF-1-7a and c) comparison plot with IRMOF-1 and IRMOF-1-C_7_. Pore size distribution was calculated by using Horvath-Kawazoe (HK) method ^[19]^ with slit pore geometry (original HK) and interaction parameter of 3.490×10^-43^ erg·cm^4^. Interaction parameter was calculated based on the polarizability and diamagnetic susceptibility of nitrogen molecules.^[20]^


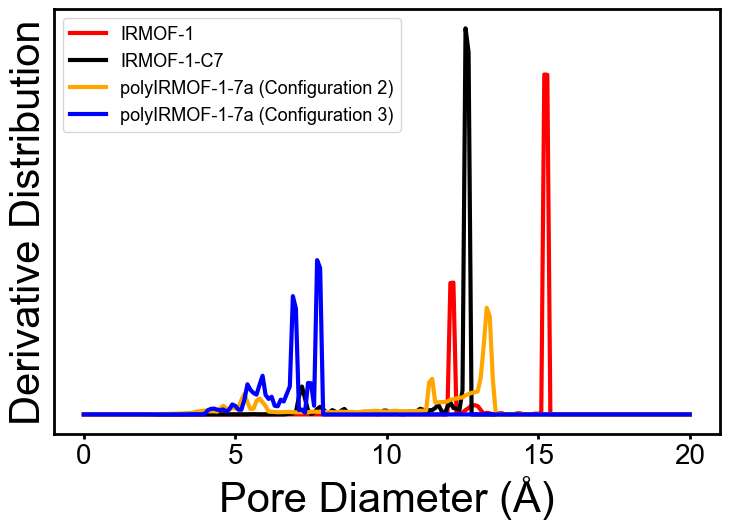


**Figure S12**. Simulated pore size distribution of IRMOF-1 (red), IRMOF-1-C_7_ (black), and polyIRMOF-1-7a in configuration 2 (orange) and configuration 3 (blue), respectively.


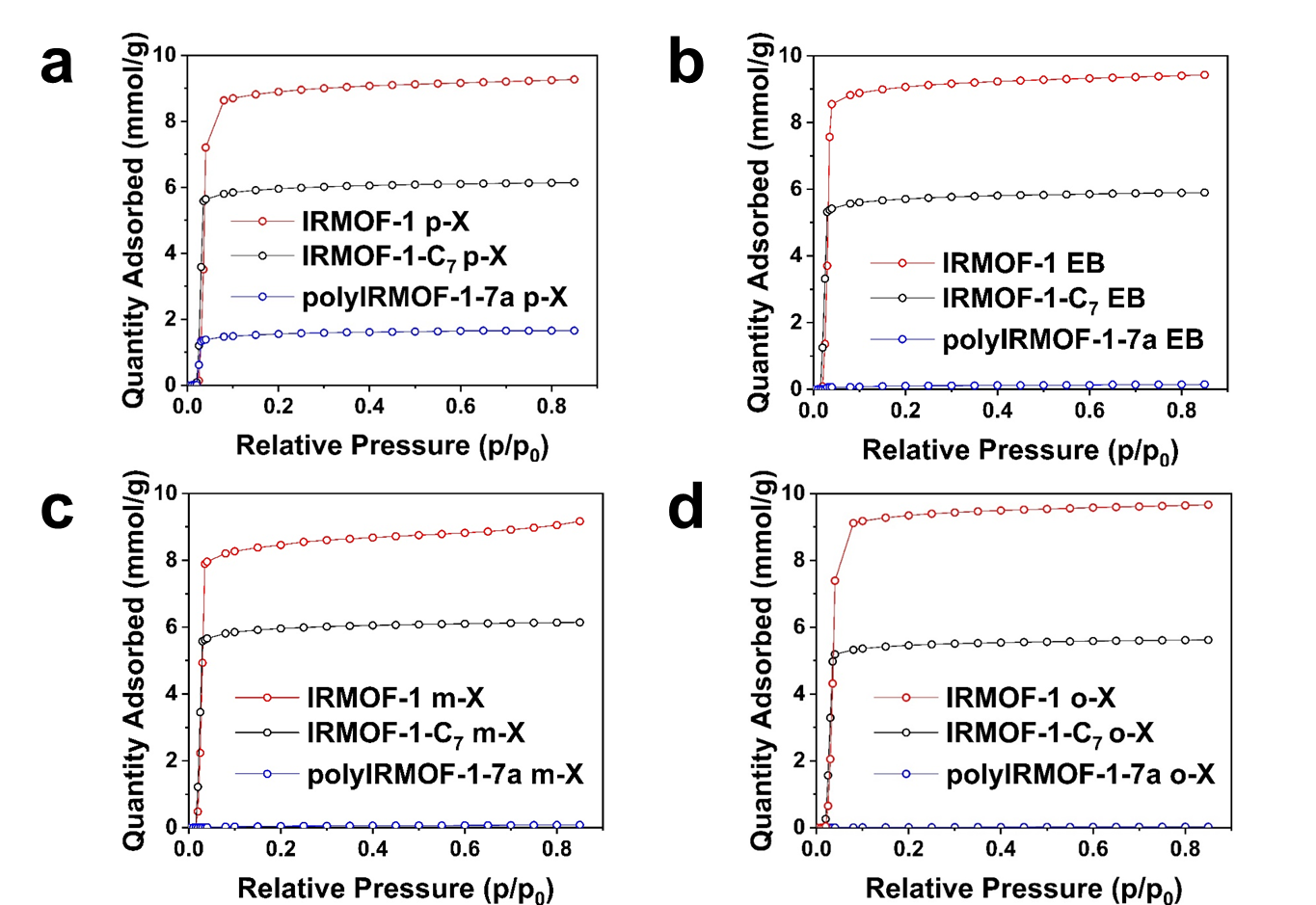


**Figure S13**. Unary vapor phase adsorption of xylene isomers in IRMOF-1 and IRMOF-1-C_7_, a) p-xylene, b) ethylbenzene, c) m-xylene and d) o-xylene.


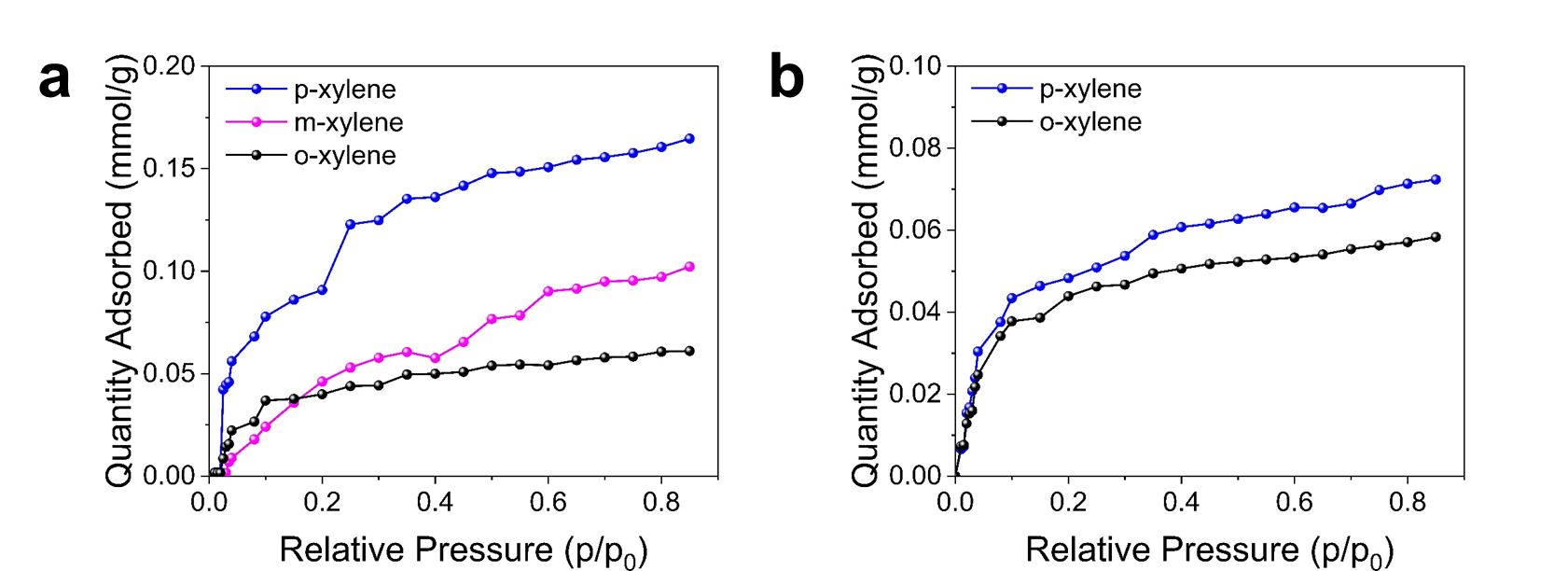


**Figure S14**. Unary vapor sorption isotherm of xylene isomers at 298K: **a)** polyIRMOF-1-5a and **b)** polyIRMOF-1-6a


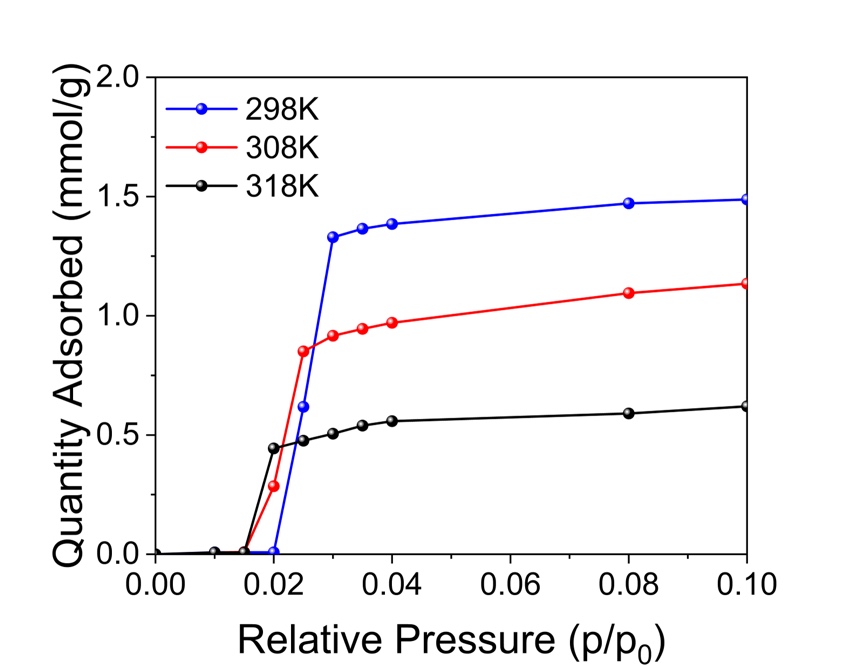


**Figure S15**. Unary vapor phase adsorption of p-xylene in polyIRMOF-1-7a at 298K, 308K and 318K


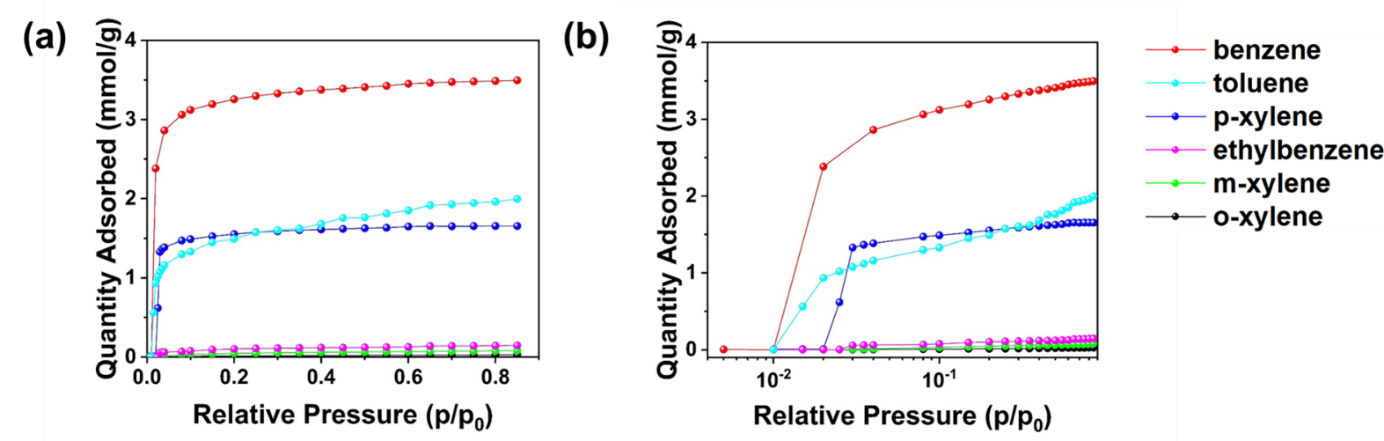


**Figure S16**. a) Unary vapor phase adsorption isotherm of polyIRMOF-1-7a at 298K for benzene (red), toluene (cyan), p-xylene (blue), ethylbenzene (magenta), m-xylene (green) and o-xylene (black) and b) logarithmic scale.





**Figure S17**. In-situ IR spectroscopy of alumina (Al_2_O_3_) with the nitrogen flow consisting of 5% of p-xylene. P-xylene was adsorbed onto the Al_2_O_3_ at 50°C for 0.5 hours to prevent condensation on the Al_2_O_3_.





**Figure S18**. Simulated IR spectrum of p-xylene using Density Functional Theory (DFT) with the B3LYP functional and 6-311G basis set.


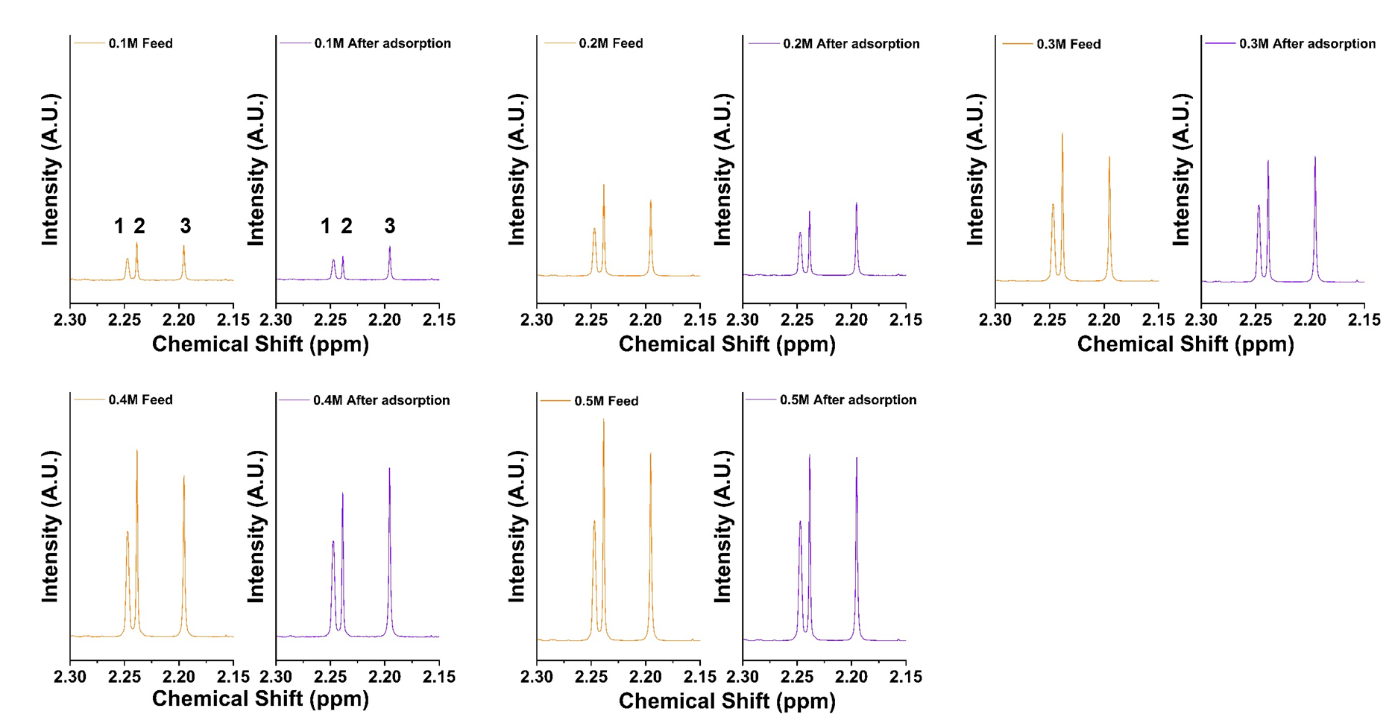


**Figure S19**. Magnified ^1^H nuclear magnetic resonance (NMR) data for ternary mixture batch adsorption of polyIRMOF-1-7a, **1**: m-xylene, **2**: p-xylene and **3**: o-xylene


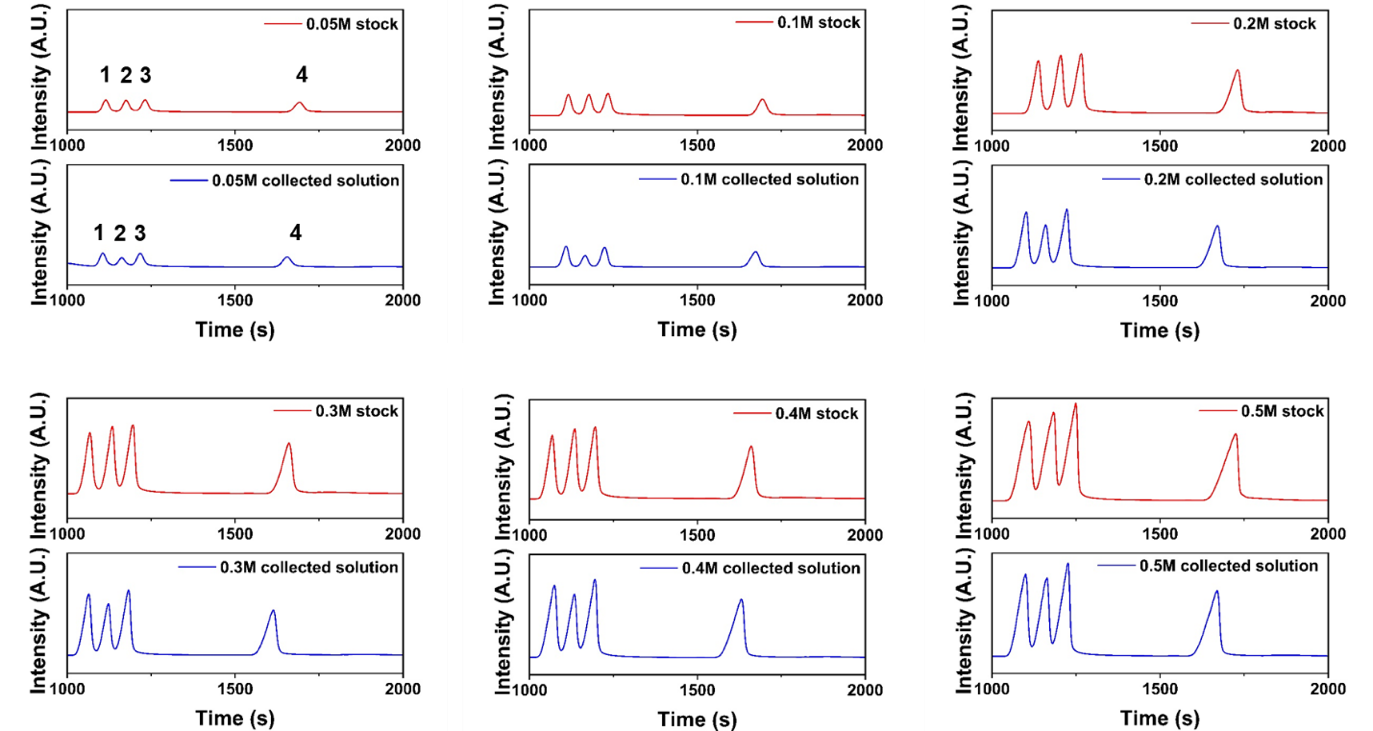


**Figure S20**. Gas chromatography data for quaternary mixture batch adsorption of polyIRMOF-1-7a, **1**: ethylbenzene, **2**: p-xylene, **3**: m-xylene and **4**: o-xylene


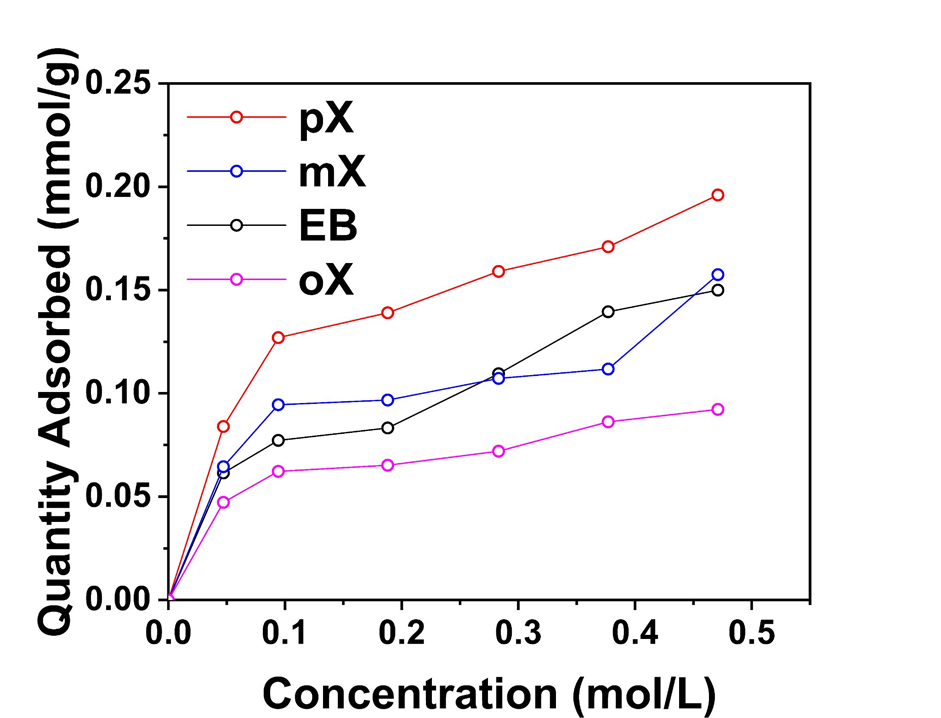


**Figure S21**. Quaternary liquid mixture batch isotherm of xylene isomers for polyIRMOF-1-5a


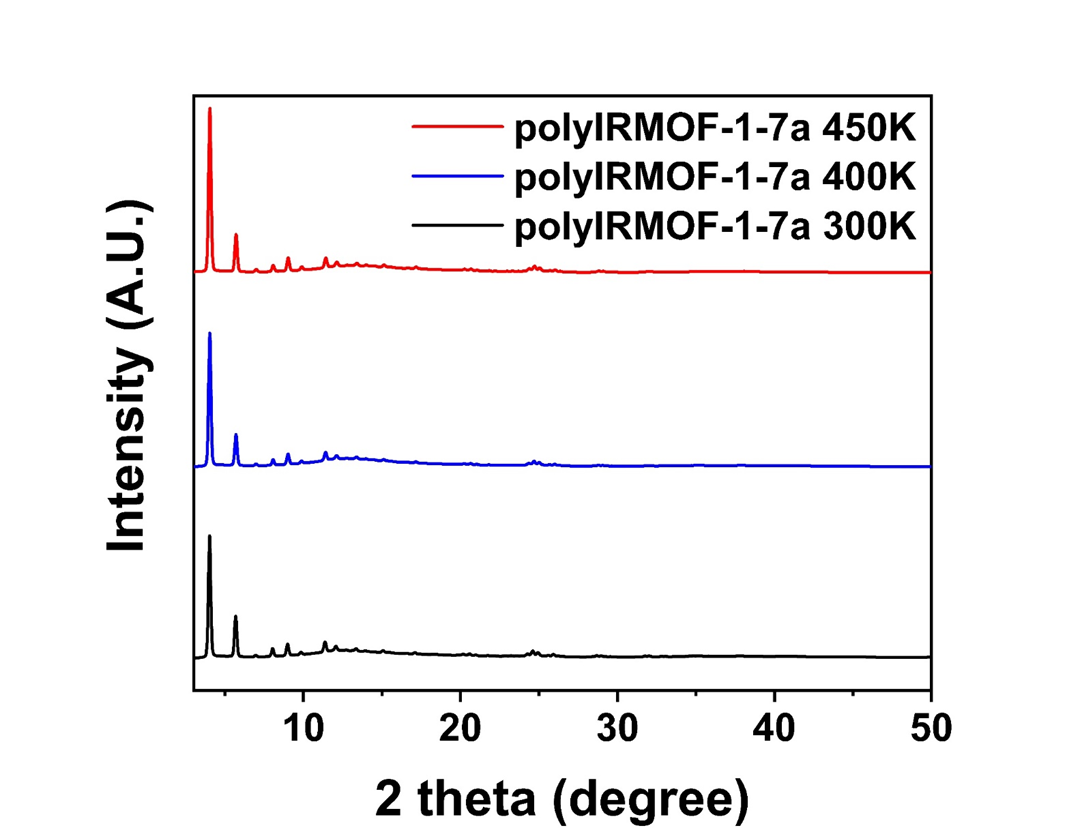


**Figure S22**. Synchrotron 2D-SMC data for polyIRMOF-1-7a at different temperature.


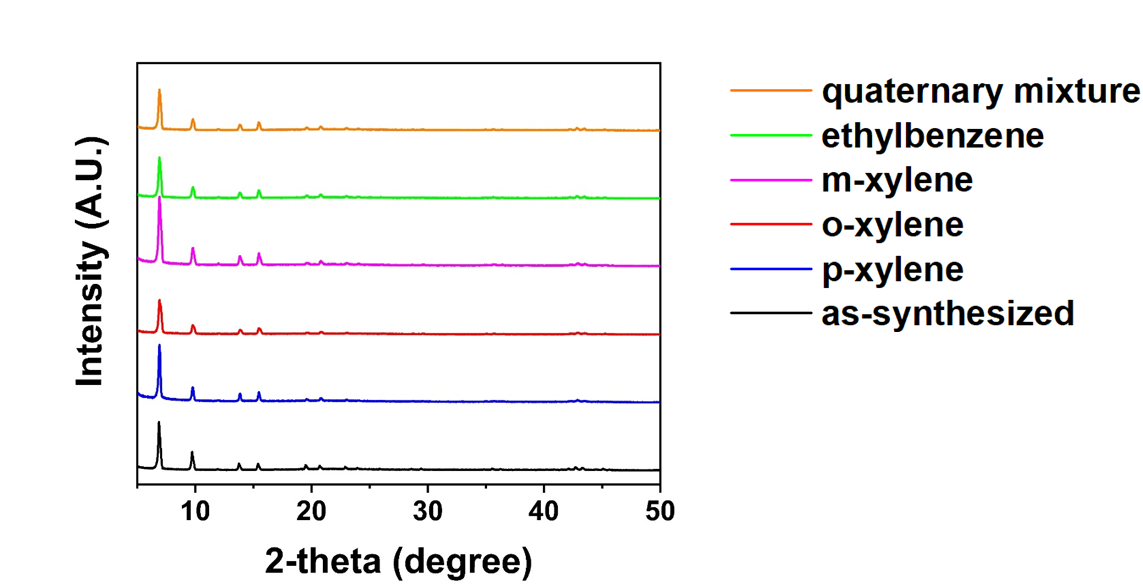


**Figure S23**. Powder x-ray diffraction (PXRD) data of pristine polyIRMOF-1-7a (black) and after quaternary mixture batch adsorption (orange) and unary vapor sorption test (green, magenta, red and blue).


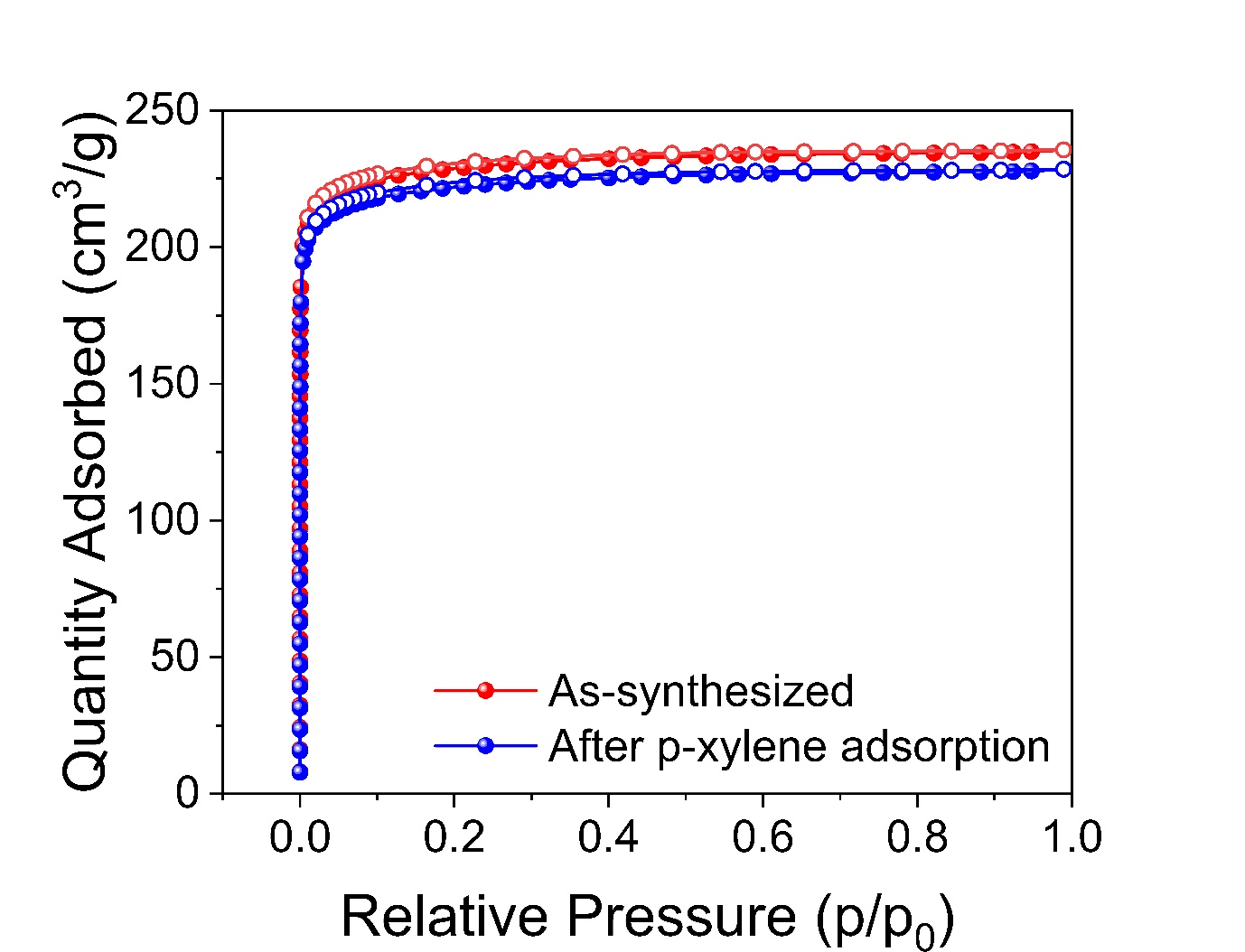


**Figure S24**. 77K nitrogen physisorption data of as-synthesized polyIRMOF-1-7a (red) and polyIRMOF-1-7a sample after the unary vapor phase adsorption of p-xylene (blue). Activation process was identical for each polyIRMOF-1-7a samples.


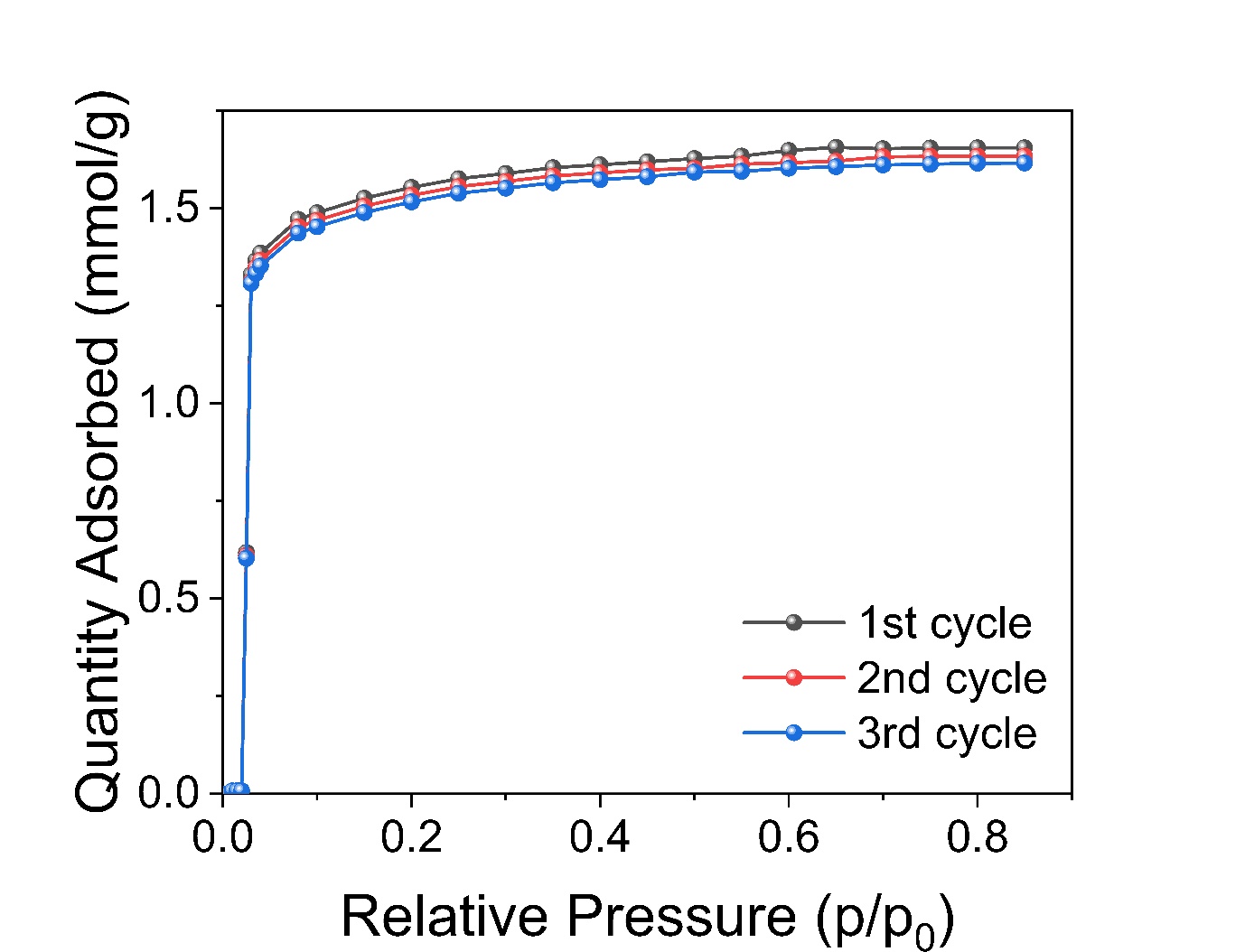


**Figure S25**. Cyclic unary vapor phase adsorption of p-xylene at 298K for polyIRMOF-1-7a. For each cycle, polyIRMOF-1-7a was activated at 75°C with nitrogen flow in organic vapor sorption apparatus (VTI-SA+).


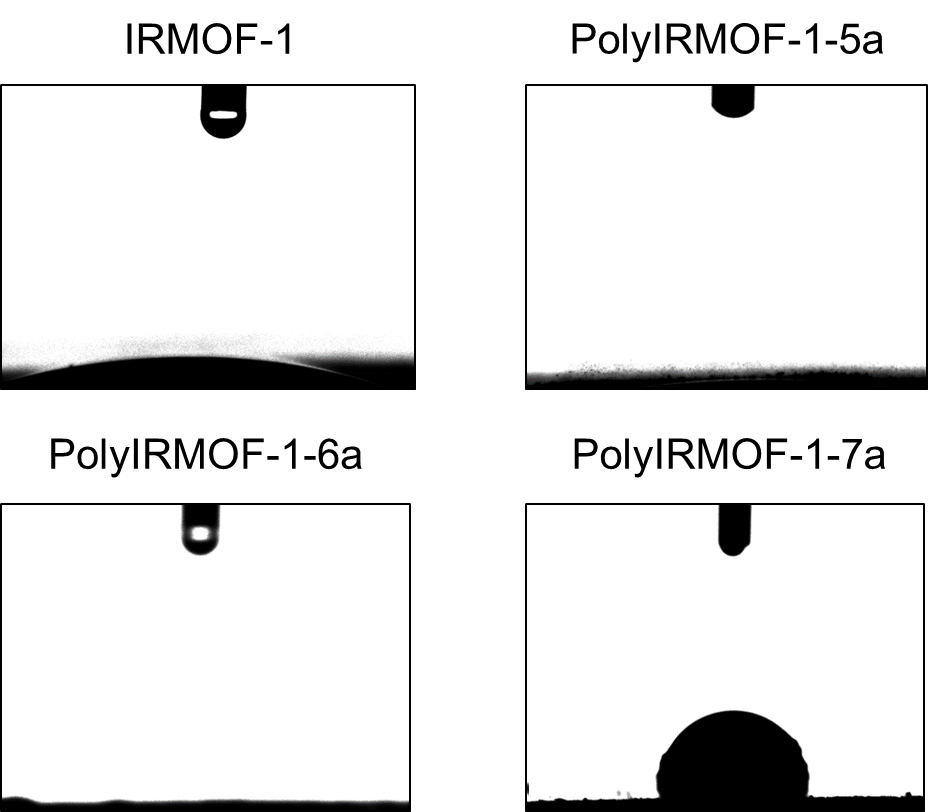


**Figure S26**. Powder contact angle measurement for each adsorbent.


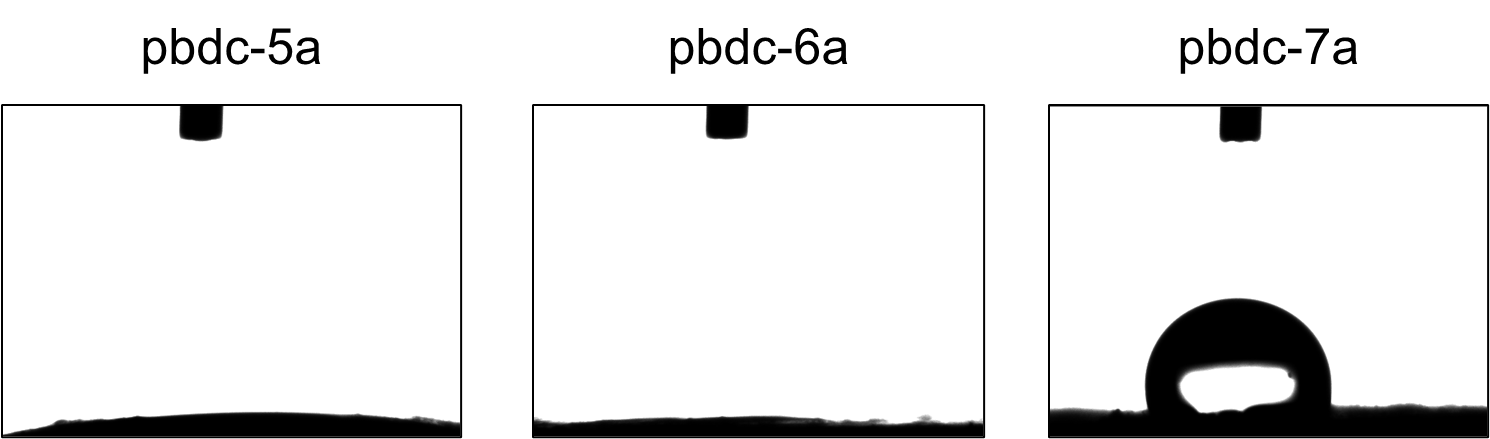


**Figure S27**. Powder water contact angle measurement for precursor polymeric ligands


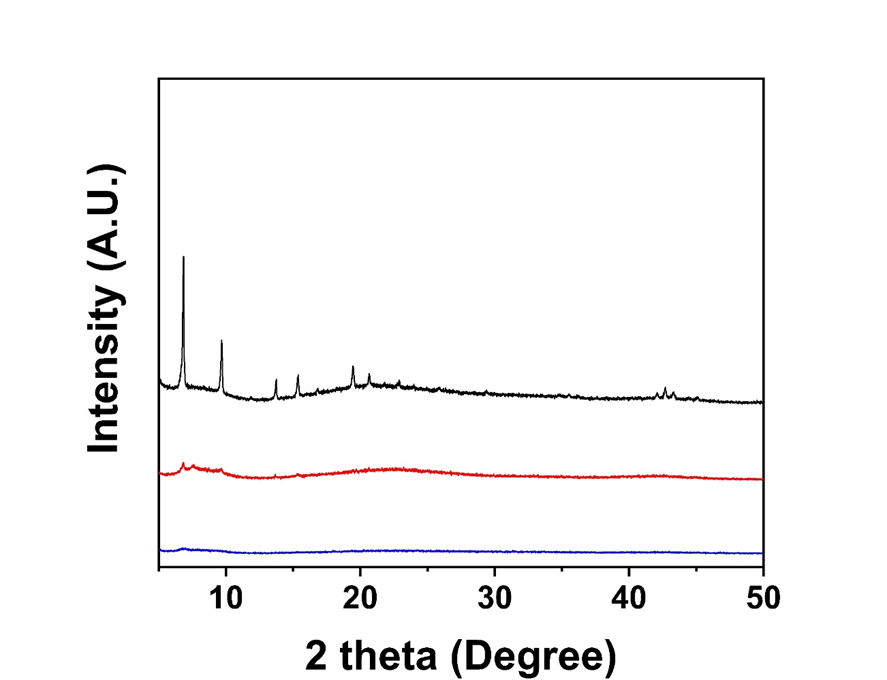


**Figure S28**. Powder x-ray diffraction (PXRD) data of pristine polyIRMOF-1-5a (black), after quaternary liquid mixture batch adsorption (red) and after unary vapor phase sorption of m-xylene (blue)


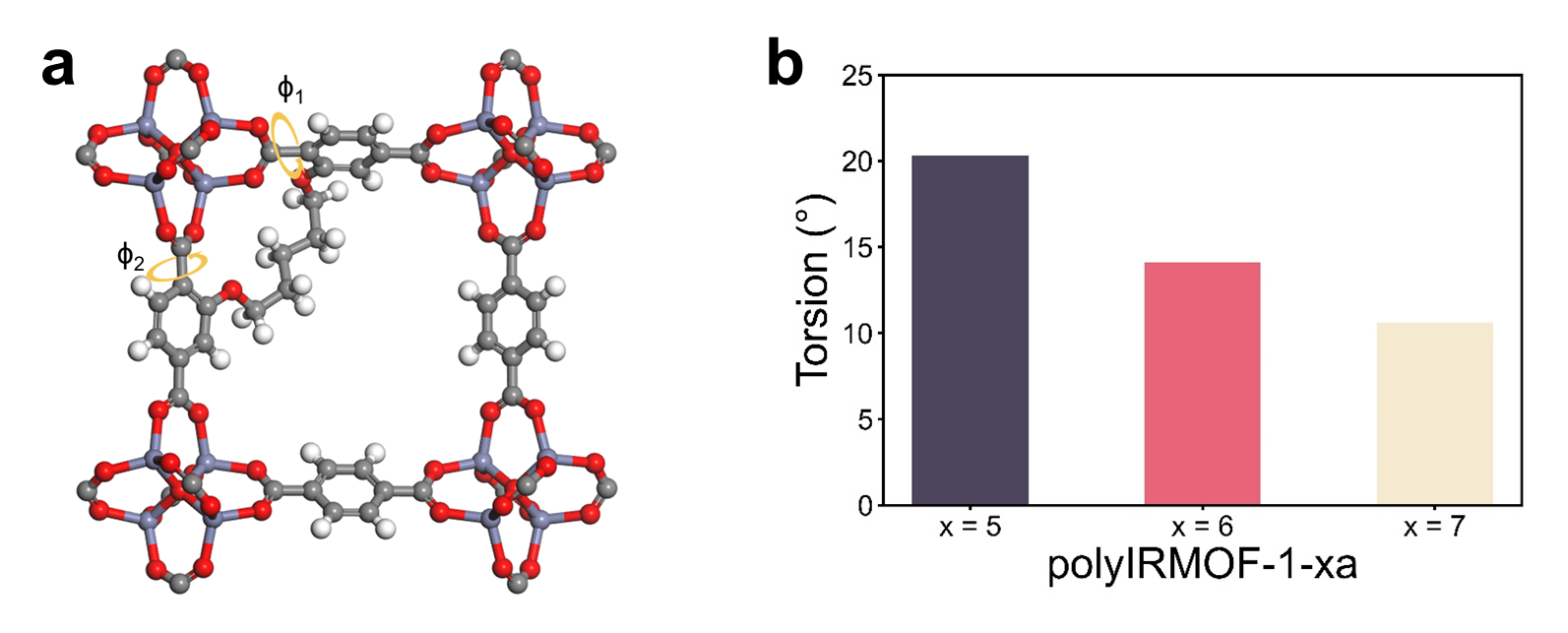


**Figure S29**. (a) Torsion on bdc ligands induced by alkyl chain. (b) Comparison of torsion exerted on bdc ligands with different length of alkyl chain. Three different configurations of each conformer were prepared and their average torsion values was obtained and compared.

**References**

[1] R. A. Dodson, J. Park, J. Kim, M. J. Cliffe, S. M. Cohen, *Inorg. Chem.* **2022**, *61*, 12284.

[2] D. Joubert, *Phys. Rev. B - Condens. Matter Mater. Phys.* **1999**, *59*, 1758.

[3] J. P. Perdew, K. Burke, M. Ernzerhof, *Phys. Rev. Lett.* **1996**, *77*, 3865.

[4] A. D. Becke, E. R. Johnson, *J. Chem. Phys.* **2005**, *123*, 0.

[5] A. K. Rappé, C. J. Casewit, K. S. Colwell, W. A. Goddard, W. M. Skiff, *J. Am. Chem. Soc.* **1992**, *114*, 10024.

[6] A. K. Rappe, W. A. G. Iii, E. Qa, *J. Phys. Chem.* **1991**, *95*, 3358.

[7] T. F. Willems, C. H. Rycroft, M. Kazi, J. C. Meza, M. Haranczyk, *Microporous Mesoporous Mater.* **2012**, *149*, 134.

[8] J. Lannoeye, B. Van de Voorde, B. Bozbiyik, H. Reinsch, J. Denayer, D. De Vos, *Microporous Mesoporous Mater.* **2016**, *226*, 292.

[9] F. Vermoortele, M. Maes, P. Z. Moghadam, M. J. Lennox, F. Ragon, M. Boulhout, S. Biswas, K. G. M. Laurier, I. Beurroies, R. Denoyel, M. Roeffaers, N. Stock, T. Düren, C. Serre, D. E. De Vos, *J. Am. Chem. Soc.* **2011**, *133*, 18526.

[10] S.-I. Kim, S. Lee, Y. G. Chung, Y.-S. Bae, *ACS Appl. Mater. Interfaces* **2019**, *11*, 31227.

[11] K. B. Idrees, Z. Li, H. Xie, K. O. Kirlikovali, M. Kazem-Rostami, X. Wang, X. Wang, T.-Y. Tai, T. Islamoglu, J. F. Stoddart, R. Q. Snurr, O. K. Farha, *J. Am. Chem. Soc.* **2022**, DOI 10.1021/jacs.2c03114.

[12] L. Yang, H. Liu, D. Yuan, J. Xing, Y. Xu, Z. Liu, *ACS Appl. Mater. Interfaces* **2021**, *13*, 41600.

[13] J. S. Wright, I. J. Vitórica-Yrezábal, S. P. Thompson, L. Brammer, *Chemistry (Easton).* **2016**, *22*, 13120.

[14] K. Jie, M. Liu, Y. Zhou, M. A. Little, A. Pulido, S. Y. Chong, A. Stephenson, A. R. Hughes, F. Sakakibara, T. Ogoshi, F. Blanc, G. M. Day, F. Huang, A. I. Cooper, *J. Am. Chem. Soc.* **2018**, *140*, 6921.

[15] L. Li, L. Guo, D. H. Olson, S. Xian, Z. Zhang, Q. Yang, K. Wu, Y. Yang, Z. Bao, Q. Ren, J. Li, *Science (80-. ).* **2022**, *377*, 335.

[16] N. Sun, S.-Q. Wang, R. Zou, W.-G. Cui, A. Zhang, T. Zhang, Q. Li, Z.-Z. Zhuang, Y.-H. Zhang, J. Xu, M. J. Zaworotko, X.-H. Bu, *Chem. Sci.* **2019**, *10*, 8850.

[17] B. Moosa, L. O. Alimi, A. Shkurenko, A. Fakim, P. M. Bhatt, G. Zhang, M. Eddaoudi, N. M. Khashab, *Angew. Chem. Int. Ed Engl.* **2020**, *59*, 21367.

[18] S. Mukherjee, B. Joarder, B. Manna, A. V Desai, A. K. Chaudhari, S. K. Ghosh, *Sci. Rep.* **2014**, *4*, 5761.

[19] G. HORV&Aacute;TH, K. KAWAZOE, *J. Chem. Eng. Japan* **1983**, *16*, 470.

[20] J. P. Ross and Olivier, *On Physical Adsorption*, J. Wiley And Sons, New York, **1964**.
